# Comparison of the composition and antiplasmodial activity of *Artemisia annua* teas using an untargeted metabolomic approach

**DOI:** 10.1371/journal.pone.0330682

**Published:** 2025-08-22

**Authors:** Agnès Aubouy, Abdou Madjid Olatoundé Amoussa, Latifou Lagnika, Nicolas Fabre, Adrien Vitrai, Catherine Claparols, Sandrine Houzé, Véronique Sarrasin, Florence Chapeland-Leclerc, Gwenaël Ruprich-Robert, Sandra Bourgeade-Delmas, Hounnankpon Yedomonhan, Charlotte Dumondin, Valérie Jullian

**Affiliations:** 1 UMR 152 PharmaDev, Université Paul Sabatier-Toulouse 3, IRD, Université de Toulouse, Toulouse France; 2 Laboratoire de Biochimie et Substances Naturelles Bioactives (LBSNB), Faculté des Sciences et Techniques (FAST), Université d’Abomey Calavi, Bénin; 3 Institut de Chimie de Toulouse, ICT UAR, Université Paul Sabatier-Toulouse III, Toulouse, France; 4 LCC-CNRS, Université de Toulouse, CNRS, Toulouse, France; 5 Université Paris Cité, IRD, MERIT, Paris, France; 6 CNR du Paludisme, AP-HP, Hôpital Bichat, Paris, France; 7 UMR 8038 CiTCoM, CNRS, Université de Paris Cité, Paris, France; 8 Laboratoire de Botanique et d’Ecologie des plantes, Faculté des Sciences et Techniques (FAST), Université d’Abomey Calavi, Bénin; Freie Universität Berlin: Freie Universitat Berlin, GERMANY

## Abstract

The use of teas made from locally cultivated *Artemisia annua* to fight malaria in remote areas where access to care is difficult is a matter of debate. This study aimed at document differences in the composition of *A. annua* teas cultivated in Benin to be sold as antimalarial teas, and in France, and their impact on antiplasmodial activity. *A. annua* teas were prepared with plants from one location in south France and from ten different plantations in Benin. Artemisinin was quantified in herbal teas with a liquid chromatography system coupled to mass spectrometry and multiple reaction monitoring detection methods. The herbal teas were tested against chloroquine-sensitive 3D7 strain of *Plasmodium falciparum* using isotopic microtest to determine IC_50_ values and calculate the concentration of artemisinin corresponding to the IC_50_ of the teas [ART(tea)_IC_50_]. Chemical profiles were determined by liquid chromatography coupled to high resolution mass spectrometry and a metabolomic analysis was performed to annotate compounds statistically linked to the antiplasmodial properties of the teas. Artemisinin content varied between 0.3 mg/L for tea with plants from France to 15.7 mg/L for teas made with plants from Benin with differences between locations. Artemisinin content was decreasing after a one-year storage of the plant for 3 localities in Benin with loss of 33%, 48% and 24% (P < 0.05). Artemisinin concentrations and antiplasmodial activity of teas were positively correlated although the comparison of ART(tea)_IC_50s_ to IC_50_ of pure artemisinin suggested that other compounds present in the tea were involved in the activity, either enhancing or limiting it. Unknown alkaloids in *A. annua* teas correlated to antiplasmodial activity were also detected. These findings suggest that *A. annua* teas deserve further studies to identify other metabolites of interest and determine their role in antiplasmodial activity in relation to other molecules, particularly artemisinin.

## Introduction

Malaria remains a deadly disease with 263 million cases and 597 000 deaths estimated in 2023. Sub-Saharan Africa accounts for a very large proportion of malaria morbidity and mortality and 76% of malarial death in this Region are children under five [1]. Treatment of malaria caused by *P. falciparum*, the most widespread and most virulent species, is based on Artemisinin-based Combination Therapies (ACTs) containing a hemisynthetic derivative of artemisinin and a partner molecule. The emergence and spread of ACT-resistant *P. falciparum* isolates is a major concern worldwide given that no effective alternative treatment is currently available [2]. Artemisinin (ART), a highly effective antimalarial molecule, was first isolated and characterized from the plant *Artemisia annua* L*.* by the Chinese chemist Youyou Tu in 1972, as the plant was part of the traditional Chinese pharmacopeia for its antipyretic properties [3]. *A. annua* plays a key role in the fight against malaria, since its production is necessary for isolating ART and developing its hemisynthetic derivatives, such as artesunate and artemether, the best-known treatments of which are available.

The use of teas made from locally cultivated *A. annua* to fight malaria in endemic areas is a topic of debate. Such a strategy could constitute an alternative antimalarial treatment for people living in remote areas where access to healthcare is limited. A certain efficacy was previously proven, both to cure and to prevent malaria [4,5]. However, a high relapse rate was observed after 14 days of treatment, suggesting a risk of under-dosing of ART, conducive to the emergence of ART-resistant parasites [6]. The variability of the chemical composition of plants depending on their environment is a well-known phenomenon which can have a significant impact on the efficacy of bioactive plants [7,8]. Other parameters influence the therapeutic effectiveness of a plant material, including plant genetic factors, drying and storage conditions. For these reasons, the WHO does not recommend the use of non-pharmaceutical form of *A. annua* [9].

During the Covid-19 pandemia, *A. annua* herbal preparations gained popularity in different parts of the world. Our study carried out in 2022 in Benin highlighted that this fame has endured, and that a chain of production, sale and consumption of *A. annua* teas exists in Benin, independently of the network of traditional practitioners [10]. In Benin, *A. annua* teas have obtained market authorization as improved traditional medicine and are sold for the treatment of uncomplicated malaria, a fatal disease for non-immune subjects if treatment is ineffective or administered too late [11]. In this context, it is extremely important to document the variability in the composition of teas and its impact on their antiplasmodial efficacy. Thus, we studied the composition of *A. annua* teas prepared with plants grown in different plantations in southern Benin Republic and France, and evaluated their *in vitro* antiplasmodial activity. Additionally, we assessed the impact of compositional variability on activity and identified potential bioactive metabolites other than ART, using untargeted metabolomic analysis.

## Materials and methods

### Plant material

**Fig 1:**
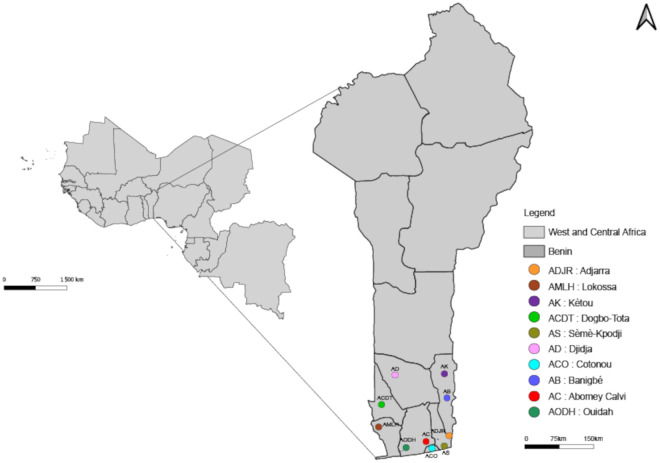
Location of the ten *A. annua* plantations in Benin that took part in the study. The maps of West Africa and Benin used for this figure come from the geoBoundaries project: Runfola, D. et al. (2020) geoBoundaries: A global database of political administrative boundaries. PLoS ONE 15(4): e0231866. https://doi.org/10.1371/journal.pone.0231866.

The plant names have been checked with https://www.worldfloraonline.org on the 6^th^. of March 2025. Aerial parts of *A. annua* were collected between September and November 2020, in ten plantations in Benin (Fig 1, Table S1 in S2 File). In each plantation, three samples were collected from different locations spaced at least 1 meter apart in the field and labelled sample_1, 2, 3. The plants were dried in a drying oven at 45°C for one week, ground, and stored at ambient temperature until further use. A voucher specimen from each plantation was botanically identified and deposited in the National Herbarium of Benin (University Abomey-Calavi, voucher numbers A003 A/HNB to A0012 A/HNB). Because we also needed *Artemisia afra* Jacq. ex Willd. for the quantification of ART, one sample of *A. afra* was collected from the Banigbé plantation, where both species *A. afra* and *A. annua* are cultivated. Post-harvest processing (drying and grinding) and tea preparation were carried out in the same way as for *A. annua*. A voucher specimen was deposited at the National Herbarium of Benin under the number A003 B/HNB.

Furthermore, two samples from the “conservatoire botanique de Soual” (Soual, France), were collected at two different time points “Soual_01” in January 2021, “Soual_02” in May 2021. The plant was dried at ambient temperature, ground, and stored at ambient temperature until further use. The samples were given to us by Dr Christel Fiorini, responsible for ‘Conservatoire Botanique Pierre Fabre’, Pierre Fabre Laboratories, Soual, France.

### Molecular analysis of plant samples

Genomic DNAs from the various *A. annua* and *A. afra* samples were extracted using the DNeasy Plant Mini Kit (Qiagen) in accordance with the manufacturer’s instructions. Approximately 100 mg of each sample was ground with liquid nitrogen in a mortar with a pestle to a fine powder, followed by further crushing with the Biorad PRECESS24 homogenizer. Amplification of the ITS1 or ITS2 regions, was performed by PCR with GoTaq polymerase (Promega), with a hybridization temperature of 56°C, according to the supplier’s recommendations, and as described by Wang [12]. The PCR amplified regions of the expected size (707 bp and 337 bp for ITS1 and ITS2 markers, respectively) were visualized on 1% agarose gel and sent for sequencing to our provider (Genewiz).

Sequence comparisons between PCR amplified regions obtained in this study and the known ITS sequences of *Artemisia* sp., retrieved from the NCBI website (http://www.ncbi.nlm.nih.gov/), have been realized using the CLUSTALW software.

### Tea preparation

Teas from *A. annua* and *A. afra* samples were prepared according to Mueller [4]. Two series of teas were prepared using *A. annua* samples collected from Benin between September and November 2020: one in February 2021 (Series 1) and one in February 2022 (Series 2). For the samples from Soual, each sample was extracted in triplicate. Briefly, 1 g (± 0.005 g) of plant was put in a tea bag and 200 mL of boiling distilled water were poured over. The beaker was covered and left at ambient temperature. The tea bag was stirred from time to time. After 15 minutes, the tea bag was squeezed and removed, and the tea was left to reach ambient temperature then filtered over cotton. Aliquots of 2 mL were stored at −20°C until use for the quantification of ART and LC-HRMS profiling for metabolomic analysis. Five of these were freeze-dried to evaluate the total concentration of the tea, and were used for the antiplasmodial assay. We verified that the composition of frozen and freeze-dried samples remained unchanged under these conditions by comparing the LC-HRMS profiles of nine samples after three years of storage at −20°C (Figs S10, S11 and S12 in S1 File).

### Quantification of ART

A standard stock solution of ART (98% purity, ref 361593, Sigma Aldrich, Saint Quentin Fallavier, France) of 0.5 mg/mL was prepared in methanol. The *A. afra* tea was diluted 1000x in milliQ water. The standard stock solution of ART was diluted in the diluted *A. afra* tea to a concentration of 5 µg/mL (solution A). Working solutions were prepared by dilution of solution A in diluted *A. afra* tea to afford the concentration range 0.5, 3.125, 5, 6.25, 12.5, 25 and 50 ng/mL for the calibration standards. Before analysis, *A. annua* teas were diluted 100x or 1000x in milliQ water to be within the linearity range and above the limit of quantification (LOQ) value.

Precision was defined as the relative standard deviation (%RSD) of the means, while accuracy was defined as the relative error (%RE) between the nominal, and the calculated concentration of ART. The limit of detection (LOD) and the LOQ were the concentrations resulting in a signal-to-noise ratio (S/N) of 3 and 10 respectively, with acceptable accuracy and precision.

Quantification of ART was done with a UPLC system coupled to a triple quadrupole-linear ion trap tandem mass spectrometry with multiple reaction monitoring detection methods. The 2 transitions 283 → 219 and 283 → 229 were used to quantify ART. The LC separation was carried out as followed: sample aliquots (10 μL) were injected onto an Acquity UPLC^TM^ BEH C18 column (1.7 µm, 130Å, 100 × 2.1 mm), fitted with a VanGuard™ C18 1.7 μm guard column (Waters, Milford, MA, USA). Column oven temperature was maintained at 40°C. Flow rate was 0.3 mL/min. The mobile phase consisted in milliQ water (solvent A) and CH_3_CN (solvent B), each containing 0.1% of formic acid. The column was equilibrated in 5% B and elution was carried out 0.5 min after injection with the following steps: 9.5 min, 95% B; 11.5 min, 95% B; 12 min, 5% B. The column was equilibrated for 2 min between injections. Detection was achieved with an Applied Biosystems Sciex QTRAP® 4500 hybrid linear ion trap triple quadrupole (LTQ) mass spectrometer (Foster City, USA) equipped with a Turbolon-Spray Interface. The instrument was operated in a positive ESI MRM mode (dwell time, 25 ms). The operating parameters were: capillary voltage, 5.5 kV; source temperature, 450°C; gas N_2_; curtain gas, 25; ion source gas 1, 50; ion source gas 2, 50; CAD medium; DP25; EP 10; CE 15; CXP 14. For MS spectra and chromatogram acquisition and exploitation, Analyst 1.6.1 software from Applied Biosystems Sciex (Foster City, USA) was used.

### LC-HRMS profiling of teas and metabolomic analysis

A 2 mL aliquot of tea was centrifuged for 5 min at 2000 g and the supernatant was analyzed using a UPLC-HRMS composed of an Ultimate 3000 (Dionex) UPLC controlled by Chromeleon Xpress 6.8 (Dionex), and a mass spectrometer LTQ Orbitrap XL (Thermo Fisher Scientific, Waltham, MA, USA). An aliquot of 10 µL of supernatant tea was injected and the chromatographic parameters were identical to those described for the quantification of ART. Spectrum were recorded using the LCQ Xcalibur 3.0 software (Thermo Fisher Scientific) in ESI + mode. The instrument settings were as follows: mass scan range m/z 100–2000 Da, capillary temperature 300 °C, sheath and auxiliary gas flow rates at 55 and 10 arbitrary units, spray voltage at 4.2 kV, source current at 100 μA, capillary and tube lens voltages set at 50 and 120 V, respectively. The mass measurement was externally calibrated before starting the experiment. Each full MS scan was followed by data dependent MS/MS analysis on the three most intense peaks using stepped collision-induced dissociation (CID) (normalized collision energy 35%, isolation width 1 Da, activation Q = 0.250). Data visualization and analysis were performed with MZmine 2.53 software [13].

For metabolomic analysis, each sample was injected three times in random order. Blanks (distilled water) and quality control (QC) samples (a mixture of all samples) were regularly added to the sequence. A first batch of analysis consisted of the first series of extraction run for the Beninese samples (90 injections), and Soual samples (18 injections). Another batch of analysis carried out 3 weeks later consisted of the second series of extraction run for the Beninese samples. A preliminary inspection was performed to check the homogeneity of the chromatograms obtained per sample and of those of the QC samples.

Raw data were processed as previously described, with no logarithmic transformation, to run multivariate analysis [Principal Component Analysis (PCA) and orthogonal partial least squares analysis (OPLS) associated with antiplasmodial activity (IC_50_, and ART(tea)_IC_50_)] on the W4M Platform (Workflow4 Metabolomics, https://workflow4metabolomics) [14]

### *In vitro* antiplasmodial evaluation

The activity against the chloroquine-sensitive *P. falciparum* strain 3D7 (from the Malaria Research and Reference Reagent Resource Center, MR4) of lyophilized herbal teas and pure ART was evaluated using the isotopic ^3^H-hypoxanthine incorporation assays, as previously described [15] with minor modifications. Briefly, *P. falciparum* parasites were maintained in O+ human erythrocytes (provided by Etablissement français du sang, EFS, Rungis, France) at 5% hematocrit and suspended in complete culture medium RPMI 1640 supplemented with 25 mM HEPES, 20 mM D-glucose, 25 mM sodium bicarbonate, 5 mM L-glutamine, and 10% AB human serum. All culture assays were conducted at 37°C, under a N_2_-enriched atmosphere (5% CO_2_, 10% O_2_, and 85% N_2_). At ring stage, *P. falciparum* cultures were highly synchronized by two consecutive treatments with 5% sorbitol (Sigma-Aldrich) in PBS (v/v) at 40 h intervals and diluted down to 0.3–0.5% parasitemia and 2% hematocrit. Freeze-dried aliquots of herbal teas were dissolved in water and then serially diluted in 96-well plates at concentrations ranging from 0.017 µg/mL to 20 µg/mL. Parasites were then dispensed and plates were incubated in the presence of 5% ^3^H-hypoxanthine (Perkin Elmer, Waltham, Massachusetts, USA) for 42 h. Scintillation counting was used to evaluate ^3^H-hypoxanthine uptake (Micro β2, Perkin Elmer, Waltham, Massachusetts, USA) and results were expressed as the inhibitory concentrations (IC_50_) defined as drug concentrations at which 50% of ^3^H-hypoxanthine incorporation was inhibited compared with drug-free controls. IC_50_ values were established by non-linear regression with ICEstimator software (http://www.antimalarial-icestimator.net/ 28 December 2021) [16]. The tests on 96-well plates were conducted in triplicate, meaning that three batches of parasites were prepared independently and distributed across three different plates.

### Calculation of the concentration of ART corresponding to the IC_50_ value of the tea, called ART(tea)_IC_50_

To determine if the presence of other compounds in the tea will increase or limit the antiplasmodial effectiveness of ART in the teas as compared to pure ART, we calculated for each tea the concentration of ART that corresponds to the IC_50_ of the tea. This value was called ART(tea)_IC_50_ and calculated as follows: ART(tea)_IC_50_ = C_ART x (IC_50_/C_Tea)

ART(tea)_IC_50_ is expressed in ng/mL

C_ART: concentration of ART in the tea, expressed in mg/L

C_Tea: Total concentration of the tea, expressed in mg/mL

IC_50_: 50% inhibitory concentration of the tea, expressed in µg/mL

### Statistical analysis

Statistical analysis was performed using GraphPad Prism software (version 9.5.1). For descriptive analysis of ART concentrations and IC_50_ values, quantitative variables were presented as the means ± standard deviations. Unpaired t-test was used to compare the groups, after the normality of the values was verified using the Shapiro-Wilk test, which is suitable for small sample sizes. Spearman correlation was used to test the nonlinear association between ART concentrations and IC_50_ values of the teas. Differences were considered significant at P < 0.05.

### Ethical considerations

The study was approved by the Research Ethics Committee of the Institute of Applied Biomedical Sciences (CER-ISBA) in Benin with authorization n°146 of 08/06/2022. The protocol was explained to the *A. annua* producers, who signed an informed consent allowing us to collect three samples from their field.

## Results

### Validation of *A. annua* identification by molecular approach

As the ITS2 marker was described as the most robust for differentiating *Artemisia* species, PCR amplifications of the ITS2 region has been first carried out on genomic DNA from all the samples listed in Fig 1. Except for one sample (*Artemisia afra*, collected in the Banigbé plantation), a fragment of the expected size (337 bp) was obtained after PCR amplification in all the samples. Sequence alignments between newly PCR amplified sequences and the known sequences of *Artemisia sp*. demonstrated unambiguously that all this samples are identical to known sequence of *A. annua* with 100% identity. By the same way, for the sample of *A. afra*, PCR amplification was realized for ITS1 marker, and sequence alignment also confirmed unambiguously that our *A. afra* sample had 100% identity to known sequence of *A. afra*. We conducted this verification to eliminate any possibility of confusion between the two species that can be mistaken for each other.

### Validation of the method for ART quantification

A linearity range between 0.5 and 50 ng/mL was first determined with ART diluted in methanol for both transitions 283 → 219 and 283 → 229 (Figs S1 and S2 in S1 File). The same linearity range was also determined with ART diluted in diluted *A. afra* teas (Figs S3 and S4 in S1 File). *A. afra* is another medicinal plant used to treat malaria, which contains no ART, but also contains flavonoids, chlorogenic acids derivatives, and coumarins, some of which are shared with *A. annua* [17]*.* We showed that the use of these calibration curves avoided matrix effect. Indeed, it was not possible to quantify directly ART in the tea. Dilution by at least a factor 100 was required to obtain a linear response (Figs S5 and S6 in S1 File). The standard addition method (avoiding matrix effect) and the method using a calibration curve made with diluted *A. afra* tea were compared for three samples (Table S2 in S2 File). The difference between the concentrations obtained by the two methods was less than 8%. Therefore, we concluded that matrix effect could be avoided by using a calibration curve made with diluted *A. afra* tea. The obtention of the validation parameters for the quantification of ART in the tea using this method is showed in the Sup. Data (Figs S3 and S4 in S1 File, Tables S3 and S4 in S2 File). LOD were determined at 1.0 and 2.5 ng/mL for the 283 → 219 and 283 → 229 transitions respectively. LOQ were determined at 2.5 and 6.25 ng/mL for the 283 → 219 and 283 → 229 transitions respectively They are summarized in the Table 1.

**Table 1:** Validation parameters for the quantification of ART in *A. annua* teas.

	*m/z* 283 → 219	*m/z* 283 → 229
Regression equation	y = 7650x + 669	y = 3270x + 1000
r^2^	0.9959	0.9947
Linear range, ng/mL	0.5-50	0.5-50
Intraday precision % min:max	4.18: 4.80	4.30: 6.76
Intraday accuracy % min:max	3.33: 5.73	5.47: 7.60
Interday precision % min:max	3.68: 9.48	0.13: −8.37
Interday accuracy % min:max	2.59: 11.43	0.40: **−14.67**
LOD, ng/mL	1.0	2.5
LOQ, ng/mL	2.5	6.25

The response was linear between 0.5 and 50 ng/mL. However, sensitivity was better for the 283 → 219 transition, as showed by the LOQ and LOD values, and the non-satisfying interday accuracy value of 14.67% for the standard at 0.5 ng/mL measured using the 283 → 229 transition. Results for both transitions are summarized in Tables S5 and S6 in S2 File. For some samples, quantification was repeated several times independently (2–4 times), and the calculated relative standard deviation was less than 14% for the 283 → 219 transition and less than 16% for the 283 → 229 transition. For all these reasons, we choose to work with values obtained using the 283 → 219 transition. Interestingly, Table S7 in S2 File shows that the values obtained using the two transitions are comparable (differences < 12%). With this method, ART concentration can be measured down to 2.5 ng/mL in a tea diluted at least 100 times, which correspond to a minimum concentration of 0.25 mg/L in the tea.

### ART concentrations differed according to the geographic origin of the plants, the series of extraction, and the harvesting period

**Fig 2:**
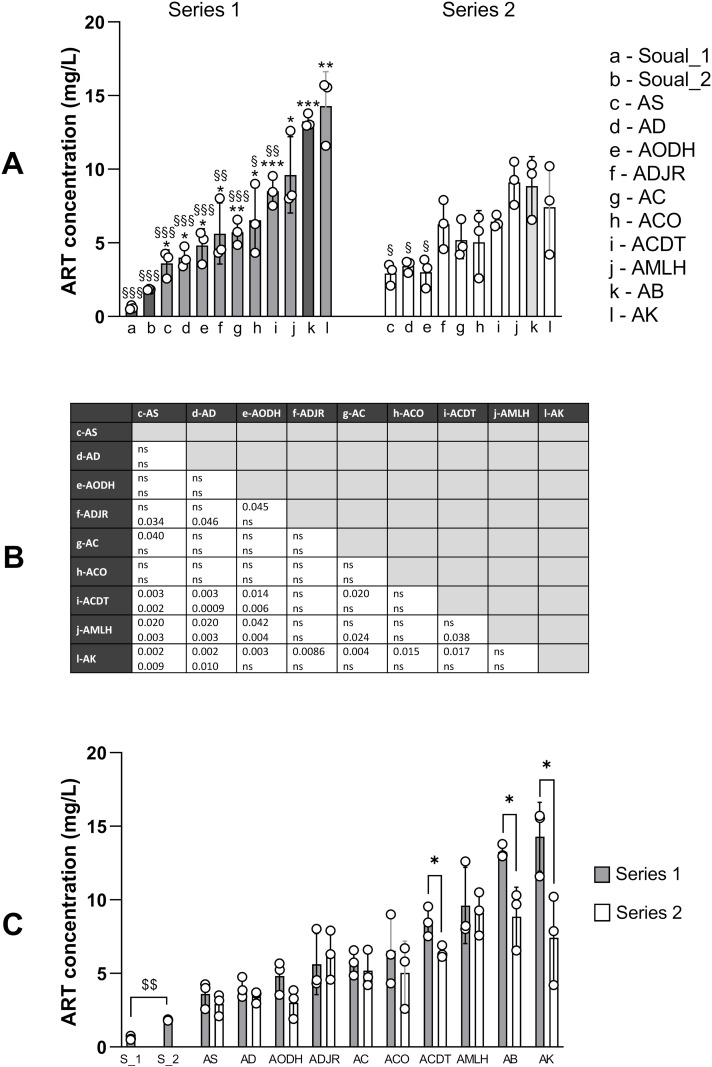
ART concentrations in herbal teas from Benin and France. (A) Concentrations were compared for each series of preparation between locations. To avoid overloading the graph, comparisons with Soual_01 and Soual_02 are symbolized by * for series 1 (the highest P value was considered between comparison to Soual_01 or Soual_02); comparisons with Banigbé (AB) are symbolized by § for both series; (B) the table shows the P values of the other comparisons between locations. (C) Concentrations were compared for each location between the two series of preparations carried out one year apart (series 1 and 2 prepared in Feb. 2021 and Feb. 2022); for Soual in France, the symbol $ indicates the comparison between two harvest periods (January and May 2021). Comparisons were carried out using a t-test. One symbol means P < 0.05, two symbols P < 0.005, three symbols P < 0.0005.

The results for ART concentration in the different teas are summarized in Table S8 in S2 File. ART concentrations ranged from 0.3 mg/L (herbal teas made with plants from Soual, France) to 15.7 mg/L for teas made with plants from Kétou (AK). For herbal teas made with plants from Benin, ART concentrations ranged from 2.6 (Sèmè-Kpodji, AS) to 15.7 mg/L. As shown on Figs 2A and 2B, for the first series of preparations, the 10 herbal teas made with plants from Benin exhibited higher ART concentrations than the two herbal teas made with plants from France (see symbol * on the Fig 2A). In addition, the herbal teas made with plants from Banigbé (AB) and Kétou (AK) had higher ART concentrations than most of the herbal teas made with plants from other localities (all localities except the herbal tea from Lokossa – AMLH). In this first series, many others differences emerged between localities with P < 0.05 or P < 0.005 (Fig 2B). For the second series of preparations (Fig 2A), significant differences in ART concentration were found between the tea from Banigbé (AB) and three others teas (Sémé-Kpodji-AS, Adjarra-AD and Ouidah-AODH) and only AS and AD teas had different ART concentrations to Kétou (AK) tea (Fig 2B). Interestingly, the two series had strikingly different profiles. This indicates that series 2 had a lower ART content than series 1, particularly in locations where the ART content was highest in series 1.

We then compared ART concentrations between the two series of tea preparation (Fig 2C). For most herbal teas (7/10), ART concentrations were similar between preparation series 1 and 2. However, for herbal teas prepared with plants from Banigbé (AB), Kétou (AK) and Dogbo (ACDT), the concentrations in the second series were lower, showing losses of 33%, 48% and 24% respectively (P = 0.02, 0.04 and 0.03, respectively). Additionally, Fig 2C presents a comparison between ART concentrations obtained during two different times of plant harvesting at the Soual site in France (Soual_01 and Soual_02). The comparison was highly significant (P = 0.0001), suggesting that harvest time is also a determining factor in the amount of ART in *A. annua* herbal teas.

**Fig 3:**
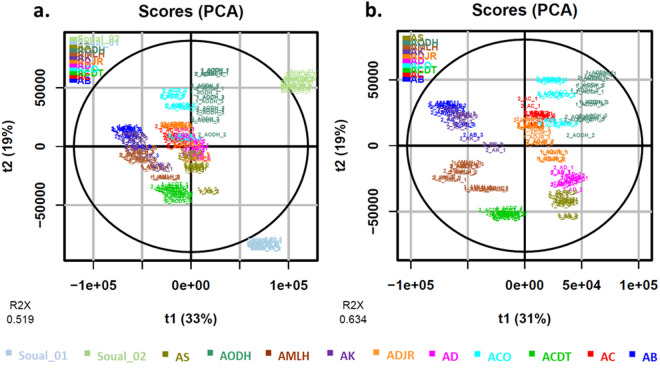
Principal component analysis (PCA) of *A. annua* herbal teas prepared with plants from different origins in Benin and France. Samples from Benin are called AS, AD, AODH, ADJR, AC, ACO, ACDT, AMLH, AB and AK. For these samples, numbers 1 and 2 refer to two series of tea preparations (Feb. 2021 and Feb. 2022). Samples from France are called Soual_01 and Soual_02 and were harvested at two different periods (Jan. 2021 and May 2021). a. PCA with French and Beninese samples. b. PCA with Beninese samples only.

In order to better understand the variability between tea samples prepared with plants of different origins, we used unsupervised metabolomic analysis on the LC-HRMS chromatograms. Principal component analysis (PCA) was initially performed with French and Beninese samples, and then with Beninese samples alone (Fig 3). To enhance statistical power, the PCA incorporated the values from both series of herbal tea preparations and, as well as the two harvesting periods for the French samples. Fig 3 shows that French samples had a different composition than Beninese samples. They appeared as outliers in Fig 3, on the right of the PCA plot, characterized by the lowest ART content. In contrast, samples from Banigbé (AB), Kétou (AK), Lokossa (AMLH) and Dogbo (ACDT), clustered on the left side of the PCA, represented the samples with the highest ART content. Indeed, when looking at variable loading, the feature at m/z = 209.1536 and rt = 8.22 min, corresponding to the base peak of ART, had one of the highest negative loading on horizontal axis. However, this is not the only clustering factor: samples from the same origin cluster together, even if they have different ART content. This can be seen with series 1 and 2 of the AK, AB and AMLH samples, for example. Furthermore, the samples from Banigbé (AB) and Kétou (AK) displayed similar profiles, despite being prepared with plants from different plantations.

As we noticed a change in the composition for the Beninese samples between the two series of tea preparation, we performed an Orthogonal Partial Least Squares-Discriminant Analysis (OPLS-DA) to compare the two batches and identify variables that may change during the storage of *A. annua* powders. The outputs of the OPLS-DA analysis are presented in Fig S7 in S1 File and the variables with the highest VIP scores are presented in Table 2. Entry 7 corresponds to the base peak of ART (m/z = 209.1536 and rt = 8.22 min), whose signal is higher in the first series, confirming that the quantity of ART decreased during the conservation of the powder. Noteworthy, entry 1 represents the signal with the highest VIP value; which is higher in the second extraction series. Entries 3, 4 and 8 are linked to it, corresponding respectively to an isotope and in source fragments. The m/z value of the MH^+^ ion associated with this signal is consistent with the molecular formula C_14_H_19_NO_2_ for the parent molecule. Entry 2 represents a MH^+^ ion corresponding to deoxyartemisinin, which has been confirmed by a comparison with a standard. Entries 5 and 9 also correspond to signals increasing during the storage of *A. annua* powders. Both signals have the same fragment ion at m/z = 130.0, which could correspond to quinoline or isoquinoline ion, and are consistent with compounds whose molecular formula are C_15_H_27_NO_4_ and C_14_H_25_NO_4_ respectively suggesting that these two compounds may share a similar structure.

**Table 2:** Features with the highest VIP values from OPLS-DA performed to discriminate the two series of teas prepared in February 2021 (series1) and February 2022 (series 2) from Beninese samples.

Entry	m/z	rt	Adduct	Molecular formula of the parent molecule	Most intense MS/MS	Hypothesis of identification	VIP score	Series with highest signal
**1**	234.1488	4.34	MH^+^	C_14_H_19_NO_2_	216.15	Unidentified	30.16	2
**2**	267.1592	8.28	MH^+^	C_15_H_22_O_4_	248.9; 239.0; 230.9; 207.0; 203.0	Deoxyartemisinin^a^	20.17	2
**3**	216.1381	4.34	Fragment	C_14_H_19_NO_2_	188.12	In source fragment of 234.1487	12.98	2
**4**	235.1520	4.34	MH^+^			Isotope of 234.1487	11.68	2
**5**	286.2010	5.98	MH^+^	C_15_H_27_NO_4_	129.97	Unidentified	10.73	2
**6**	181.1222	6.99	MH^+^	C_11_H_16_O_2_	163.0; 145.0; 107.0; 93.0	Unidentified	10.64	2
**7**	209.1537	8.22	Fragment	C_15_H_22_O_5_		ART^a^	10.49	1
**8**	188.1433	4.34	Fragment	C_14_H_19_NO_2_		In source fragment of 234.1487	10.38	2
**9**	272.1854	5.44	MH^+^	C_14_H_25_NO_4_	130.06	Unidentified	8.96	2

^a^Identified with a standard

### The variability in plantation location, plant storage time and harvesting period had an impact on the antiplasmodial activity of *A. annua* teas

**Fig 4:**
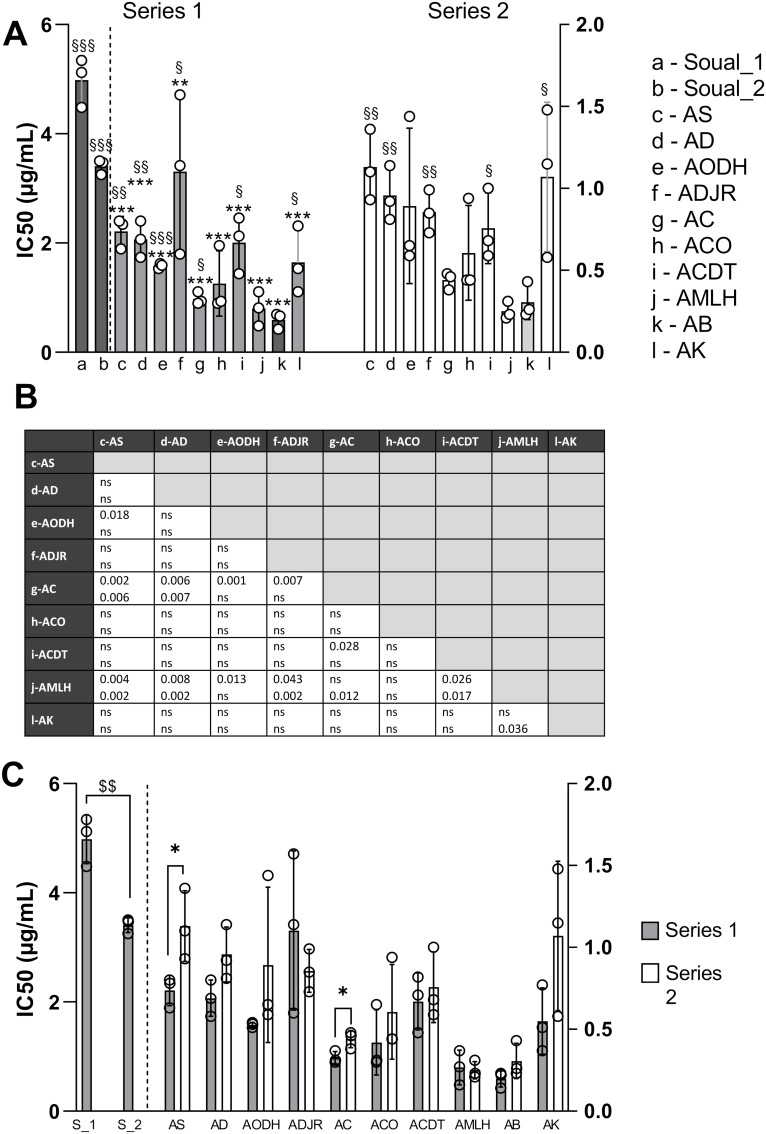
IC_50_ values of herbal teas from Benin and France. The y-axis on the left is for Soual_01 and Soual_02, the one on the right is for all the other localities in the two series. (A) IC_50_ values were compared for each series of preparation between locations. To avoid overloading the graph, comparisons with Soual_01 and Soual_02 are symbolized by * for series 1 (the highest P value was considered between comparison to Soual_01 or Soual_02); comparisons with Banigbé (AB) are symbolized by § for both series; (B) the table shows the P values of the other comparisons between locations. (C) IC_50s_ were compared for each location between the two series of preparations carried out one year apart (series 1 and 2 prepared in Feb. 2021 and Feb. 2022); for Soual in France, the symbol $ indicates the comparison between two harvest periods (January and May 2021). Comparisons were carried out using a t-test. One symbol means P < 0.05, two symbols P < 0.005, three symbols P < 0.0005.

Next, we compared *in vitro* antimalarial activities of *A. annua* teas by location, by tea preparation series, and by harvesting period for the French samples. The results are summarized in Table S8 in S2 File. For Beninese herbal teas, mean 50% inhibitory concentration (IC_50_) values ranged from 0.2 µg/mL for the herbal tea from Banigbé (AB) of the first series to 1.3 µg/mL for the tea from Sémé-Kpodji (AS) of the second series (Fig 4A, Table S8 in S2 File). In comparison, IC_50_ values obtained with *A. annua* teas from France were higher with 3.5 and 5.0 µg/mL for Soual_01 and Soual_02, respectively. As shown on Fig 4A, for the first series of tea preparation, the comparisons of IC_50s_ of herbal teas from Benin with those from Soual in France were all highly significant (P < 0.001). For both preparation series, Banigbé (AB) and Lokossa (AMLH) herbal teas exhibited lower IC_50_ values than herbal teas from other localities in Benin (P < 0.05 to P < 0.0005), except for the AMLH/ACO comparison. Other significant comparisons between localities for the two series are detailed in the table (Fig 4B).

Fig 4C highlights the impact of the preparation series on the IC_50_ values of the two herbal teas from Sémé-Kpodji (AS) and Abomey Calavi (AC). Mean values rose from 0.74 to 1.13 µg/mL for AS and from 0.33 to 0.44 µg/mL for AC between series 1 and 2. Fig 4C, also shows that IC_50_ values obtained during two different times of plant harvesting at the Soual site in France (Soual_01 and Soual_02) were significantly different (P = 0.004). As for ART concentration in plant, this indicates that harvest time is a determining factor in antiplasmodial activity. These results suggest that ART concentration in plant and antimalarial efficacy are related.

### The ART concentration of *A. annua* teas was not the only factor determining their antimalarial activity *in vitro*

**Fig 5:**
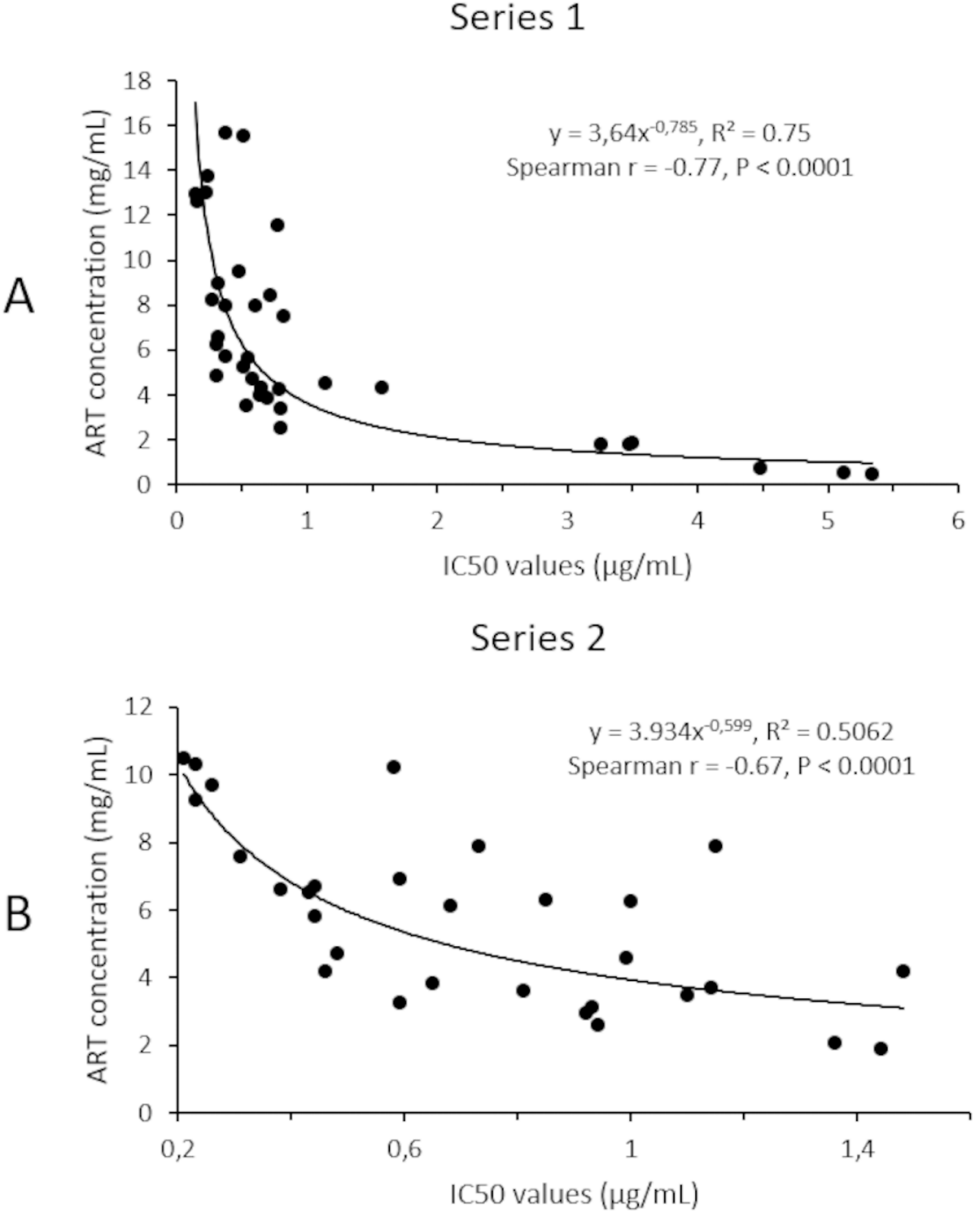
ART content as a function of IC_50_ of the teas. The curve is modelled as a power curve (Excel software) for the first (A) and the second series (B). Spearman correlation test was used to test the association between values.

To find out if ART concentration of *A. annua* teas was a determining factor in their antiplasmodial activity, we studied correlations between ART concentrations in teas and IC_50_ values. The relation between the two variables could be modelled as power curves for the two series. Equations were: y = 3.640x^—0.785^ and y = 3.934x^—0.599^ with R^2^ values of 0.75 and 0.51 for series 1 and 2 respectively, indicating that the two variables were highly correlated (Fig 5). Spearman correlation tests also resulted in r values of −0.77 and −0.67 for series 1 and 2 (P < 0.0001), showing the association between ART concentrations and IC_50s_.

However, it is interesting to note that the increasing order of ART concentrations by locality presented in Fig 2 was not the same for the IC_50_ values presented in Fig 4. In particular, the herbal tea obtained from Kétou (AK) plants had the highest ART content following the first series of herbal tea preparations, unlike the herbal teas from Lokossa (AMLH), Abomey Calavi (AC) and Cotonou (ACO). However, the AK herbal tea of the first series had higher IC_50_ values than the AMLH, AC and ACO herbal teas, suggesting that the other molecules present in the *A. annua* teas are involved in the activity, either enhancing or limiting it.

**Fig 6:**
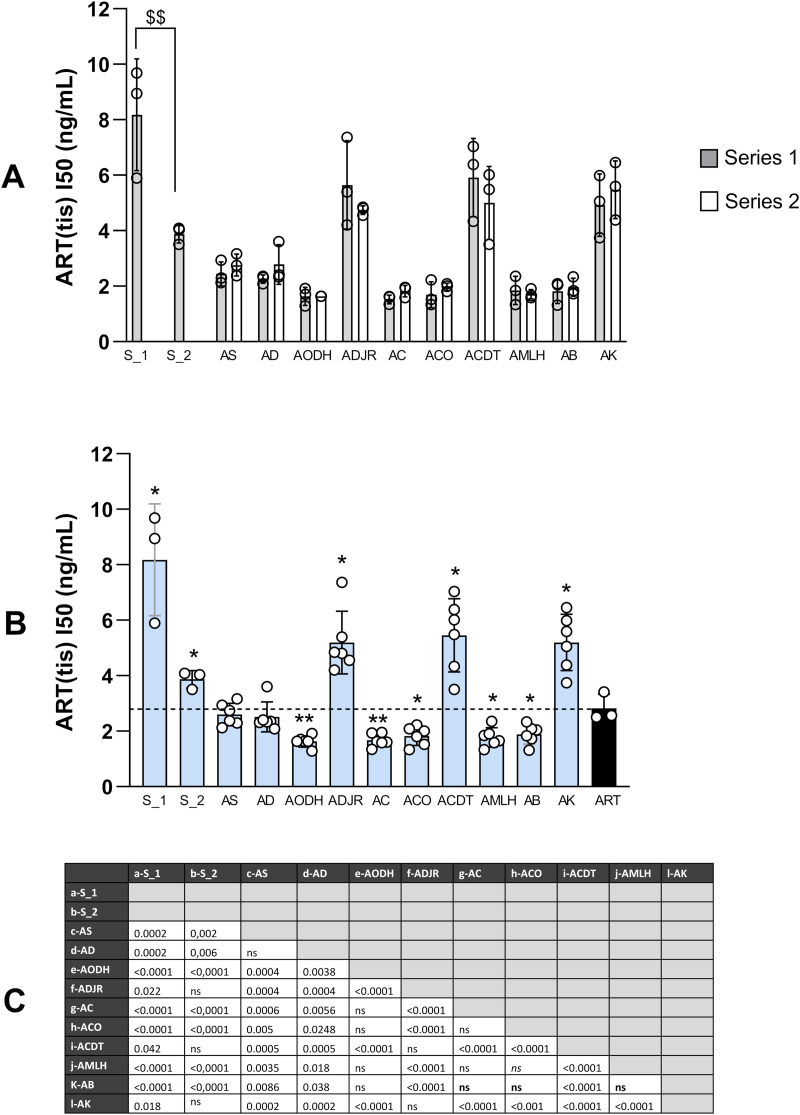
IC_50_ values based only on the ART contained in *A. annua* teas (ART(tea)_IC_50_) from Benin and France. (A) ART(tea)_IC_50s_ were compared for each location between the two series of preparations carried out one year apart (series 1 and 2 prepared in Feb. 2021 and Feb. 2022); for Soual in France, the symbol $ indicates the comparison between two harvest periods (January and May 2021). (B) ART(tea)_IC_50s_ were compared for each series of preparation between locations. Since the values were similar between the two series of preparation, the values from both series were considered for this comparison. * shows the comparisons to the IC_50_ values of pure ART; (C) the table shows the P values of the other comparisons between locations. Comparisons were carried out using a t-test. One symbol means P < 0.05, two symbols P < 0.005, three symbols P < 0.0005.

To explore this issue, we first calculated the concentration of ART corresponding to the IC_50_ value of the tea, named ART(tea)_IC_50_ (Table S8 in S2 File). The difference between this value and the IC_50_ of pure ART indicates if other molecules in the tea contribute significantly to antiplasmodial activity, enhancing it if ART(tea)_IC_50_ < IC_50_ of pure ART, limiting it if ART(tea)_IC_50_ > IC_50_ of pure ART. Fig 6 highlights the variation of this value according, as before, to the series of preparation, the location of plantations and the harvest period for the French plants (Soual_01 and Soual_02). As shown on Fig 6A, ART(tea)_IC_50_ mean values varied between 1.67 ng/mL (5.77 nM) for tea with plants from Ouidah (AODH) to 5.46 ng/mL (19.34 nM) and 8.18 ng/mL (28.97 nM) for teas with plants from Dogbo (ACDT) and from Soual (Soual_01), respectively. The comparison for each locality by series 1 and 2 did not show any significant differences (Fig 6A). However, for French teas as for IC_50_ values, the harvesting period in May led to lower value for ART(tea)_IC_50_ than in January (P = 0.01) (Fig 6A). Since the values were similar between the two series of preparation, the values from both series were considered for the comparison by locality (Figs 6B and 6C). ART(tea)_IC_50_ of the different teas were first compared to IC_50_ value of pure ART. Teas from Ouidah (AODH), Abomey Calavi (AC), Cotonou (ACO), Lokossa (AMLH) and Banigbé (AB) obtained lower ART(tea)_IC_50_ values compared to those of pure ART (mean IC_50_ = 2.82 ng/mL or 9.99 nM), suggesting that the molecules, other than ART, contained in these herbal teas act synergistically or additively to enhance their *in vitro* antimalarial activity. In contrast, Soual, Adjarra (ADJR), Dogbo (ACDT) and Kétou (AK) *A. annua* teas had higher ART(tea)_IC_50_ values than pure ART, suggesting that their composition limits the activity of the ART contained in the herbal teas. Many other differences were found between plantation locations (Fig 6A), including the significant differences between ART(tea)_IC_50_ values of teas prepared with plants from Soual_01 (harvest period January) and all Beninese teas.

### Identification of putative antiplasmodial metabolites in *A. annua* teas

To delve deeper the issue of the chemical composition of *A. annua* teas and its impact on their *in vitro* antimalarial activity, we performed OPLS analysis using the IC_50_ values as Y input. The outputs of the OPLS-DA analysis are presented in Fig S8 in S1 File. The variables with the highest VIP score are presented in Table 3. Entries 2 and 4 indicated that Arteannuin B was negatively correlated to activity. The following signals showed positive correlation with antiplasmodial activity: Entries 9 and 16 showed up signals of ART. Entries 3 and 5 represent an unknown alkaloid and deoxyartemisin, which were also linked to the ageing of the plant samples (Table 3). Entry 15 corresponds to an unknown alkaloid as well. Aminoacids (Entries 1, 6 and 10) could also contribute to activity, as well as flavonoids (entry 13).

**Table 3:** Features with the highest VIP values from OPLS performed using the IC_50_ value as Y input with all samples.

Entry	m/z	rt	Adduct	Molecular formula of the parent molecule	Most intense MS/MS	Hypothesis of identification	VIP score	Correlation with activity
**1**	116.0704	0.95	MH^+^	C_5_H_9_NO_2_	98.0; 88.0	Proline^a^	13.1	POS
**2**	266.1752	7.68	[M+NH4]^+^	C_15_H_20_O_3_	**248.8**; 231.0; 213.1; 189.0; 185.1; 176.9	Arteannuin B^a^	13.0	NEG
**3**	234.1488	4.34	MH^+^	C_14_H_19_NO_2_	216.15	Unidentified	12.8	POS
**4**	249.1486	7.68	MH^+^	C_15_H_20_O_3_	**231.0**; 213.0; 189.0; 185.0; 176.9	Arteannuin B^a^	12.3	NEG
**5**	267.1592	8.28	MH^+^	C_15_H_22_O_4_	**248.9**; 239.0; 230.9; 207.0; 203.0	Deoxyartemisinin^a^	10.7	POS
**6**	166.0860	2.17	MH^+^	C_9_H_11_NO_2_	**149.0**; 148.0; 131.1; 103.0; 93.0	Phenylalanine^a^	10.2	POS
**7**	237.1483	6.21	[M+H-H_2_O]^+^	C_14_H_22_O_4_	**220.9**; 207.0; 177.1; 151.2; 133.0; 89.0	Unidentified	9.8	POS
**8**	265.1433	6.05	[M+H-H_2_O]^+^	C_15_H_22_O_5_	247.0; **237.0**; 229.0; 177.0	Unidentified	9.6	NEG
**9**	209.1537	8.22	In source fragment	C_15_H_22_O_5_	191.0; 173.0; **151.0**; 139.0	ART^a^	9.6	POS
**10**	205.0971	3.22	MH+	C_11_H_12_N_2_O_2_	187.90	Tryptophane^a^	9.4	POS
**11**	235.1692	9.25	MH+	C_15_H_22_O_2_	217.0; 161.1; 159.0	Unidentified Not artemisinic acid^a^	8.5	POS
**12**	231.1380	7.67	[M+H-H_2_O]^+^	C_15_H_20_O_3_	215.0; **205.1**; 159.1	Arteannuin B^a^	8.4	NEG
**13**	361.0919	6.57	MH^+^	C_18_H_16_O_8_	346.0; 345.0; **327.8**; 311.0	Chrysosplenol D^b^	7.8	POS
**14**	235.1328	6.24	MH^+^	C_14_H_18_O_3_	No data	Unidentified	7.8	NEG
**15**	244.1444	4.00	MH^+^	C_14_H_17_N_3_O	226.0; 112.0; 95.0	Unidentified	7.7	POS
**16**	283.1541	8.22	MH^+^	C_15_H_22_O_5_	265.0; **247.1**;	ART^a^	7.4	POS

^a^Comparison with standards;

^b^Comparison with published MSMS data with Fu et al. (2020) Phytochemical analysis and geographic assessment of flavonoids, coumarins and sesquiterpenes in *Artemisia annua* L. based on HPLC-DAD quantification and LC-ESI-QTOF-MS/MS confirmation. Food Chem. 2020;312:126070. https://doi.org/10.1016/j.foodchem.2019.126070

**Fig 7:**
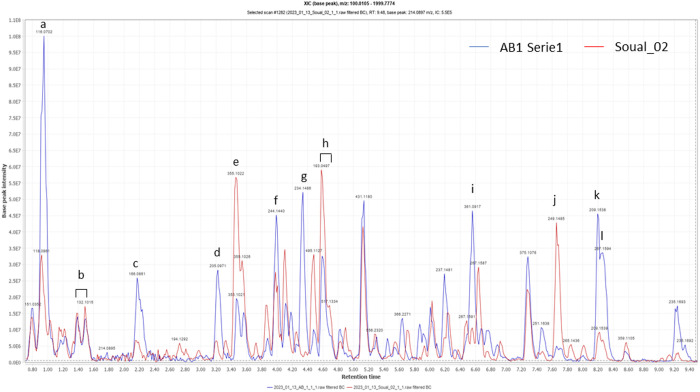
LC-HRMS chromatograms of samples AB_1 (series 1) and Soual_02. Main features described in Tables 2–4 are highlighted. a: proline, b: leucine/isoleucine, c: phenylalanine, d: tryptophan, e: chlorogenic acid, f: unknown alkaloid and 5-feruoylquinic acid, g: unknown alkaloid, h: dicaffeoylquinic acid (mixture of isomers), i: chrysosplenol D, j: arteannuin B, k: ART, l: deoxyartemisinin.

The same OPLS analysis was conducted using ART(tea)_IC_50_ as Y input. The outputs of the OPLS-DA analysis are presented in Fig S9 in S1 File, and the features with the highest VIP values are presented in Table 4. This analysis shows features corresponding to aminoacids (entries 1, 3 and 5), deoxyartemisinin (entry 10), and the unidentified alkaloids (entries 6 and 12) previously detected in Tables 2 and 3. Four features corresponding to chlorogenic acid and derivatives were also identified (entries 11, 4, 7 and 9). Among these, dicaffeoylquinic acids were identified by comparison of their signals with 3,4-; 3,5- and 4,5-dicaffeoylquinic acids standards. Due to the close retention times under our chromatographic conditions, the three isomers could not be fully discriminated, therefore the signal in entry 7 represents a mixture of these 3 isomers. Fig 7 highlights the different peaks corresponding to most of the features described in Tables 2–4 on the LC-HRMS chromatograms of one sample from Benin (AB_1) and one sample from France (Soual_02).

**Table 4:** Features with the highest VIP values from OPLS performed using ART(tea)_IC_50_ as Y input with all samples.

Entry	m/z	rt (min)	Adduct	Molecular formula of the parent molecule	Most intense MS/MS	Hypotesis of identification	VIP score	Correlation with activity
**1**	132.1016	1.46	MH^+^	C_6_H_13_NO_2_	114.0; 69.1	Leucine/isoleucine^a^	12.91	POS
**2**	237.1483	6.21	MH^+^	C_14_H_20_O_3_	**220.9**; 207.0; 177.1; 151.2; 133.0; 89.0	Unidentified	11.80	POS
**3**	116.0704	0.95	MH^+^	C_5_H_9_NO_2_	98.1; 88.1	Proline^a^	11.00	POS
**4**	517.1336	4.69	MH^+^	C_25_H_24_O_12_	499.0	Dicaffeoylquinic acid ^a^^,^^c^	11.15	POS
**5**	166.0860	2.17	MH^+^	C_9_H_11_NO_2_	**149.0**; 148.0; 131.1; 103.0; 93.0	Phenylalanine^a^	10.96	POS
**6**	244.1444	4.00	MH^+^	C_14_H_17_N_3_O	**226.0;** 112.0; 95.0	Unidentified	10.52	POS
**7**	531.1494	5.18	MH^+^	C_26_H_26_O_12_	512.9	Caffeoylferuloylquinic acid ^b^^,^^c^	9.64	POS
**8**	205.0970	3.22	MH^+^	C_11_H_12_N_2_O_2_	187.9	Tryptophane^a^	9.25	POS
**9**	369.1180	4.13	MH^+^	C_17_H_20_O_9_	**176.96**; 145.07	5-Feruloylquinic acid^b^	9.13	POS
**10**	267.1591	8.28	MH^+^	C_15_H_22_O_4_	**248.9**; 239.0; 230.9; 207.0; 203.0	Deoxyartemisinin^a^	9.10	POS
**11**	355.1023	3.52	MH^+^	C_16_H_18_O_9_	162.9	Chlorogenic acid^a^	8.69	POS
**12**	234.1488	4.34	MH^+^	C_14_H_19_NO_2_	216.1	Unidentified	8.39	POS

^a^Identified with standards;

^b^Identified by comparison of data with Carbonara et al (2012) Phytochemical analysis of a herbal tea from *Artemisia annua* L. J Pharm Biomed Anal. 2012;62:79–86. https://doi.org/10.1016/j.jpba.2012.01.015.

^c^Mixture of isomers.

## Discussion

This study, conducted using plants from Benin and France, aimed to investigate the variability in the chemical composition of *A. annua* teas based on the location of the plantations, the storage time of the plants, and the harvest period. The study also examined the impact of this variability on the *in vitro* antimalarial activity of the teas and identified putative active metabolites.

The LC-MS/MS quantification method for ART used in this study was adapted from that developed by Suberu et al. [18,19], which reported LOD and LOQ values of 0.13 and 0.41 ng/mL respectively. Others published methods based on LC-MS/MS described LOD and LOQ values of 1.5 and 5 ng/mL, similar to those obtained in this study [20,21]. The quantity of ART in the tea was variable, as already observed in previous studies. This variability depends on the variety of *A. annua* used, as some hybrids are developed to maximize ART production, which is typically low in wild type plants [22]. ART content is also influenced by geographical location and climatic condition [23,24]. The recipe used in this work, 5 g of plant for 1 L of water, was based on that described by Mueller et al. for clinical trials. Their teas were prepared with the Artemis hybrid known for its high content in ART, grown for this purpose in Democratic Republic of Congo or in Germany. The teas contained 12 mg/L and 47 mg/L of ART respectively [4,6]. Similarly, Silva et al. reported ART concentrations of 40–46 mg/L in teas prepared under comparable conditions using a high ART content *A. annua* variety developed by the UNICAMP University in Brazil [25]. Mouton et al. also compared the amount of ART in teas prepared from 16 samples of *A. annua* of diverse origins, and found concentration ranging from 8.36 to 117.2 mg/L in teas prepared with 9 g of plant for 1 L of water [26] (corresponding to 4.18 and 58.6 mg/L with 5 g of plant for 1 L of water, [6]). Therefore, the concentrations of ART measured in this work are in the lower range compared to those already published. Unfortunately, information about the varieties of *A. annua* cultivated in the Beninese plantations was unavailable. However, according to PCA (Fig 3), they are rather homogenous. Series 2 extractions performed one year after series 1 showed significant ART losses in teas prepared with plants from Dogbo (ACDT), Banigbé (AB) and Kétou (AK) (Fig 2). These findings align with WHO data from 2001 which reported ART losses of 12,87% over six months and 32.76% over 12 months storage [27]. Similarly, in this study losses in ART ranged from 24% to 48% for the affected samples. Again, in 2006, WHO warned against a storage under high humidity and temperature, which renders raw vegetal material unsuitable for ART extraction after one year [28]. Simonnet et al. observed the same phenomenon when monitoring ART content of dried *A. annua* powder. However, they demonstrated that entire dry leaves can be stored at 20 or 30°C for up to one year without any loss of ART [29]. It is important to point out here that our previous survey in Benin revealed that *A. annua* herbal teas are sold as powder in tea bags with an expiry date of three years from the date of packaging [10]. This is far too much in the light of our findings and WHO data. Table 2 highlights the most significant features obtained from an OPLS-DA comparing the global composition of series 1 and 2. These results confirmed ART degradation over time. Furthermore, we found that an alkaloid (molecular formula C_14_H_19_NO_2_) and deoxyartemisinin were the most discriminant features between the 2 groups, being higher in series 2. Therefore, ART could be converted in these two molecules during storage. Deoxyartemisinin has been characterized in *A. annua* for a long time, and is also one of the phase I metabolites of ART *in vivo* [30]. Czechowski et al. also observed an accumulation of deoxyartemisinin during *A. annua* drying (14 days at 40°C), without concurrent ART loss, attributing this to a post-harvest conversion of hydroperoxydated dihydroartemisinic acid (DHAAOOH) into deoxyartemisinin [31].

Regarding the *in vitro* antiplasmodial activity, it was clearly correlated with the concentration of ART in the teas, regardless of the preparation series of the teas. The curve of IC_50_ as a function of the concentration of ART could be modelled as a power curve (Fig 5) which is coherent with the fact that ART is the principal active component of the tea. One of the reasons for the semi-synthesis of ART derivatives is its low bioavailability [32,33]. Interestingly, Räth et al. demonstrated through a pharmacokinetic study that ART derived from the consumption of *A. annua* herbal tea was present in the plasma much more rapidly than when absorbed through capsules containing only ART. This faster bioavailability is attributed to the presence of plant constituents that enhance the solubility of ART [34]. It has been demonstrated that *A. annua* tea inhibits the activity of the hepatic cytochromes CYP2B6 and CYP3A4, thereby preventing the degradation of ART in the liver. *A. annua* tea could also prevent the transcription and translation of these cytochromes [35]. In addition, the chemical constituents of *A. annua* herbal tea may enhance the antimalarial effects of ART. In our study, some teas precisely displayed low IC_50_ values, i.e., high antiplasmodial activity, but had lower content in ART than other teas, such as the teas from Abomey Calavi (AC) and Cotonou (ACO). The fact that others compounds present in the tea may enhance the *in vitro* antiplasmodial activity of ART has been previously studied [18,25,26,36] and one tool used to evidence this is to calculate the concentration of ART corresponding to the IC_50_ value of the tea, called here ART(tea)_IC_50_, and compare it to the IC_50_ of pure ART. If ART(tea)_IC_50_ is lower than the IC_50_ of pure ART_,_ other compounds may contribute to the activity of the tea. In this study, we observed that ART(tea)_IC_50_ was lower than IC_50_ of pure ART for Ouidah (AODH), Abomey Calavi (AC), Cotonou (ACO), Lokossa (AMLH) and Banigbé (AB) samples, while it was higher for the Soual (both harvest time), Kétou (AK), Dogbo (ACDT), and Adjarra (ADJR) samples, indicating that other components of these teas may have a detrimental effect on the *in vitro* antiplasmodial activity. Such detrimental effect has never been reported before. De Donno et al. reported ART(tea)_IC_50_ values three time lower than the IC_50_ value of ART for tea made from *A. annua* cultivated in Italy and tested against D10 and W2 *P. falciparum* strains. Similarly, with an *A. annua* tea made from an ART high yielding variety, Suberu et al. reported that their tea was 3–7 times more potent that ART alone on HB3 and Dd2 strains respectively. Mouton et al. showed no significant differences for teas made from a collection of 16 different samples of *A. annua* from different varieties and origins, when tested on 3D7 strains. The same result was observed by Silva et al. with 3 samples of an ART high content *A. annua* variety from Manaus, and tested on 3D7 and K1 strains.

The hypothesis that ART is more effective when taken as a tea of *A. annua*, that is, combined with the other molecules present in the tea, is a topic of debate. Our results, together with those already published, demonstrate that this hypothesis may be true for certain plants characterized by their variety, origin, storage time, and harvest period, but the opposite may also be true. There are seminal studies showing that casticin [37] and chrysosplenol D [38], two flavonoids present in the tea, enhance the *in vitro* antiplasmodial activity of ART. However, Czechowski et al. concluded that “flavonoids do not contribute to antimalarial activity in whole leaf extracts”. This team compared the *in vitro* antiplasmodial activity of a wild *A. annua* plant and a genetically modified plant without flavonoids but producing the same amount of ART, and found no difference [39]. Suberu et al. showed that compounds present in the tea like sesquiterpenes, flavonoids, phenolic acid, caffeoylquinic acids may have synergetic, additive or antagonistic effect, depending on their ratio with ART and the *P. falciparum* strain used (CQ resistant or sensitive) [18].

These previous studies were focused on the study of known and targeted metabolites, and their capacity to enhance activity of ART *in vitro*. We deemed it relevant to model LC-MS data with untargeted metabolomic tools like OPLS to determine the link between the variation of LC-MS signals and the variation of biological activity, and uncover yet unknown metabolites potentially linked to the antiplasmodial activity of the tea. To the best of our knowledges, untargeted OPLS analysis has been used once with *in vivo* antimalarial activity of different extracts of *A. annua*, and led to the identification of several polymethoxylated flavones together with sesquiterpenes, like 5α-hydroperoxy-eudesmane-4(15),11-diene and arteannuin B, linked with antimalarial activity [40]. Another study linking by hierarchical cluster analysis (HCA) *in vitro* antiplasmodial activity of several extracts of *A. annua* and their compositions determined by GC-MS, identified various volatile compounds and deoxyartemisinin as positively correlated with the activity [41]. Our results of the OPLS analysis with IC_50_ as the response are summarized in Table 3, where the signals with the highest VIP scores are presented. For the sesquiterpenes, signals of ART (entries 9 and 16), deoxyartemisinin (entry 5) and an unidentified compound, which is not artemisinic acid (entry 11), contributed positively to activity. Deoxyartemisinin itself has a low antiplasmodial reported activity (IC_50_ > 10 µM on 3D7 strain) [42], and its link with the antiplasmodial activity of *A. annua* extracts was reported once [41]. An unidentified isomer of ART (entry 8) and arteannuin B (entries 2, 4 and 12) were correlated with a lower antiplasmodial activity. Arteannuin B has a weak antiplasmodial activity compared to ART, but has been previously identified to contribute positively to *in vitro* and *in vivo* antimalarial activity [18,43]. Indeed, for biogenetic reasons, its quantity in *A. annua* is negatively correlated to the quantity of ART [22], and this could explain its negative correlation with the activity of the tea. It should be noted that the same phenomenon may also apply to compounds that are positively correlated with activity. This may be because their quantity is positively linked to the quantity of ART, rather than because they affect antiplasmodial activity. The possibility of such indirect correlations poses a limitation to the use of OPLS for the search for new bioactive compounds in *A. annua*. Entry 13 shows that chrysosplenol D was positively correlated with activity, which is coherent with previous findings (vide supra). Surprisingly, our study highlights that several aminoacids (proline, phenylalanine, tryptophan, entries 1, 6 and 10) may contribute to antiplasmodial activity, which has not been previously reported. However, it has already been reported that short peptides including phenylalanine residues, exhibit antimalarial activity, likely by interfering with the activity of parasitic enzymes such as plasmepsin II, which is involved in hemoglobin degradation, or aminoacyl tRNA synthetase [44,45]. Interestingly, some alkaloids not previously reported in *A. annua* are positively correlated with activity (entries 3 and 14). We also modeled the link between variation of composition and variation of the ART(tea)_IC_50_ (Table 4), and this highlighted the correlation of chlorogenic acid and derivatives (entries 4, 7, 9, 11), to low values of ART(tea)_IC_50_. Suberu et al. have already reported interaction between ART, chlorogenic acid and chlorogenic acid derivatives, which may be additive, synergetic or antagonistic, depending on their ratio with ART and the strain of *P. falciparum*. These compounds showed a tendency to be synergetic at high dose on CQ-sensitive strains and antagonistic at low dose on CQ-resistant strains. The authors attributed this antagonistic interaction to the antioxidant properties of these compounds [18]. This is coherent with our results on 3D7, a chloroquine sensitive strain.

Noteworthy, the three OPLS of this study highlighted the potential role of unreported alkaloids in *A. annua*. Feature 1 in Table 2 (C_14_H_19_NO_2,_ rt = 4.34 min) appeared also in Table 3 (entry 3) and Table 4 (entry 12). Its molecular formula was the same as rupestine G and rupestine K, previously reported in *Artemisia rupestris* L. [46] although their structures were not compatible with the fragmentation pattern observed for the unknown compound. Feature 15 in Table 3 (C_14_H_17_N_3_O, rt = 4.00 min) appeared in Table 4 (entry 6). Two alkaloids, tentatively identified as quinoline or isoquinoline appeared in Table 2 (entries 5 and 9). Alkaloids are rarely mentioned in the studies on *A. annua* and their presence in the tea is yet unreported. Alkylamides can be found in Asteraceae (*Echinacea* genus), but have not been detected in this study. Interestingly, toxic pyrrolizidines alkaloids have been reported in *Artemisia capillaris* Thunb. [47] and *Artemisia scoparia* Waldst. & Kit. [48]. One sample of *A. annua* among 386 samples of others herbs used in TCM was tested for its content of these molecules, and the amount detected (11 µg/kg) was considered low and not a threat to human health [48]. A review dealing with alkaloids in the genius *Artemisia* reports only 8 alkaloids previously described in *A. annua*. Three are purine alkaloids, three are indole alkaloids, and the others two are dipeptides [49]. Alkaloids are known for their wide biological activities, as well as their potential toxicity. Isolation and identification of the alkaloids present in the teas of *A. annua* teas is therefore a major challenge.

## Conclusion

In the context of *A. annua* production for local consumption of antimalarial herbal teas, our results reveal that the teas prepared with Beninese plants present variability in their composition, and especially in their ART content, depending on the location of the plantations. Furthermore, we confirm that ART content decreases with storage duration. These findings emphasize the need for a better identification of *A. annua* varieties available on the market. Packaging should include harvest date, and shorter shelf lives should be recommended.

Further studies are also necessary to define a number of quality criteria for *A. annua* herbal teas sold as anti-malarial treatments, particularly regarding the minimum ART content for proven anti-malarial activity. Although ART is not the sole factor responsible for the activity, it is currently the most reliable chemical marker available. Defining this minimum amount would require extremely costly phase 1 and 2 clinical trials. Considering the WHO standards for efficacy (less than 10% total failure rate), achieving official approval for the use of these teas for the treatment of uncomplicated malaria seems challenging (Mueller et al. reported 77% efficacy rate at Day 7 with teas containing 47 mg of ART per litre, more than 20 years ago).

Our study also confirmed others findings showing that the claim of a better antiplasmodial activity of ART taken in a *A. annua* tea, rather than alone, thanks to the synergy of other compounds cannot be generalized, at least *in vitro*. Additionally, our results also highlighted the presence of unknown alkaloids in *A. annua* teas, which are worth identifying and studying to determine their antiplasmodial activity as well as their toxicity. Furthermore, potential contribution of amino-acids and yet unknown sesquiterpenes to the antiplasmodial activity deserves further investigations.

## Supporting information

S1 File Supporting figures Fig S1 Calibration curve for 283 → 219 transition. Solutions of ART in methanol. Fig S2 Calibration curve for 283 → 229 transition. Solutions of ART in methanol. Fig S3 Calibration curve for 283 → 219 transition. Solutions of ART in *A. afra* tea diluted 1000 times. Fig S4 Calibration curve for 283 → 229 transition. Solutions of ART in *A. afra* tea diluted 1000 times. Fig S5 Calibration curves for 283 → 219 transition. Comparison between dilution in methanol, in *A. Afra* tea, in *A. afra* tea diluted 100 times, in *A. afra* tea diluted 1000 times. Fig S6 Calibration curves for 283 → 229 transition. Comparison between dilution in methanol, in *A. Afra* tea, in *A. afra* tea diluted 100 times, in *A. afra* tea diluted 1000 times. Fig S7 Result of the OPLS-DA analysis discriminating series 1 and series 2. Fig S8 Result of the OPLS analysis with IC_50_ value as Y input. Fig S9 Result of the OPLS analysis with ART(tea)_IC_50_ value as Y input. Fig S10 LC-HRMS chromatograms of lyophilized tea dissolved in water (in red) and frozen tea (in blue) for 3 samples of series 1 (AB2, AODH1, AS1). Fig S11 LC-HRMS chromatograms of lyophilized tea dissolved in water (in red) and frozen tea (in blue) for 3 samples of series 2 (AB1, AC2, ACO2). Fig S12 LC-HRMS chromatograms of lyophilized tea dissolved in water (in red) and frozen tea (in blue) for 3 samples of series 2 (ADJR1, AMLH2, AS2).
(DOCX)

S2 File Supporting tables. Table S1 Location and date of sample collection, date of herbal tea preparation. Table S2 Comparison between results obtained with standard addition method, and with the use of a calibration curve made in *A. afra* tea diluted 1000 times, for three samples of *A. annua* tea (Soual_01_1, Soual_01_2, Soual_01_3) diluted 100 times. Table S3 Intraday variation for two standard solutions (5 and 12.5 ng/mL of ART in *A. afra* tea diluted 1000 times). Table S4 Interday variation for the standard solutions (0.5, 3.125, 5, 6.25, 12.5 ng/mL of ART in *A. afra* tea diluted 1000 times). Table S5 Results of determination of C_ART in *A. annua* teas using the 283 → 219 transition. Table S6 Results of determination of C_ART in *A. annua* teas using the 283 → 229 transition. Table S7 Comparison of the results for both transitions for the determination of C_ART in the teas expressed in mg/mL. Table S8: Summary of the results obtained for the 50% inhibitory concentration of the teas on 3D7 strain of *P. falciparum* (IC_50_), the concentration of ART in the teas (C_ART), the total concentration of the teas (C_Tea) and the concentration of ART corresponding to the IC_50_ of the teas [ART(tea)_IC_50_].
(DOCX)
