# Supplementary material for: Comparison of the composition and antiplasmodial activity of Artemisia annua teas using an untargeted metabolomic approach
Source: PLoS One. 2025 Aug 22;20(8):e0330682. doi: 10.1371/journal.pone.0330682 (PMC12373170; doi:10.1371/journal.pone.0330682)
Supplement: S1 File — Fig S1 Calibration curve for 283 → 219 transition. Solutions of ART in methanol. Fig S2 Calibration curve for 283 → 229 transition. Solutions of ART in methanol. Fig S3 Calibration curve for 283 → 219 transition. Solutions of ART in A. afra tea diluted 1000 times. Fig S4 Calibration curve for 283 → 229 transition. Solutions of ART in A. afra tea diluted 1000 times. Fig S5 Calibration curves for 283 → 219 transition. Comparison between dilution in methanol, in A. Afra tea, in A. afra tea diluted 100 times, in A. afra tea diluted 1000 times. Fig S6 Calibration curves for 283 → 229 transition. Comparison between dilution in methanol, in A. Afra tea, in A. afra tea diluted 100 times, in A. afra tea diluted 1000 times. Fig S7 Result of the OPLS-DA analysis discriminating series 1 and series 2. Fig S8 Result of the OPLS analysis with IC50 value as Y input. Fig S9 Result of the OPLS analysis with ART(tea)_IC50 value as Y input. Fig S10 LC-HRMS chromatograms of lyophilized tea dissolved in water (in red) and frozen tea (in blue) for 3 samples of series 1 (AB2, AODH1, AS1). Fig S11 LC-HRMS chromatograms of lyophilized tea dissolved in water (in red) and frozen tea (in blue) for 3 samples of series 2 (AB1, AC2, ACO2). Fig S12 LC-HRMS chromatograms of lyophilized tea dissolved in water (in red) and frozen tea (in blue) for 3 samples of series 2 (ADJR1, AMLH2, AS2). (DOCX) [file pone.0330682.s001.docx]

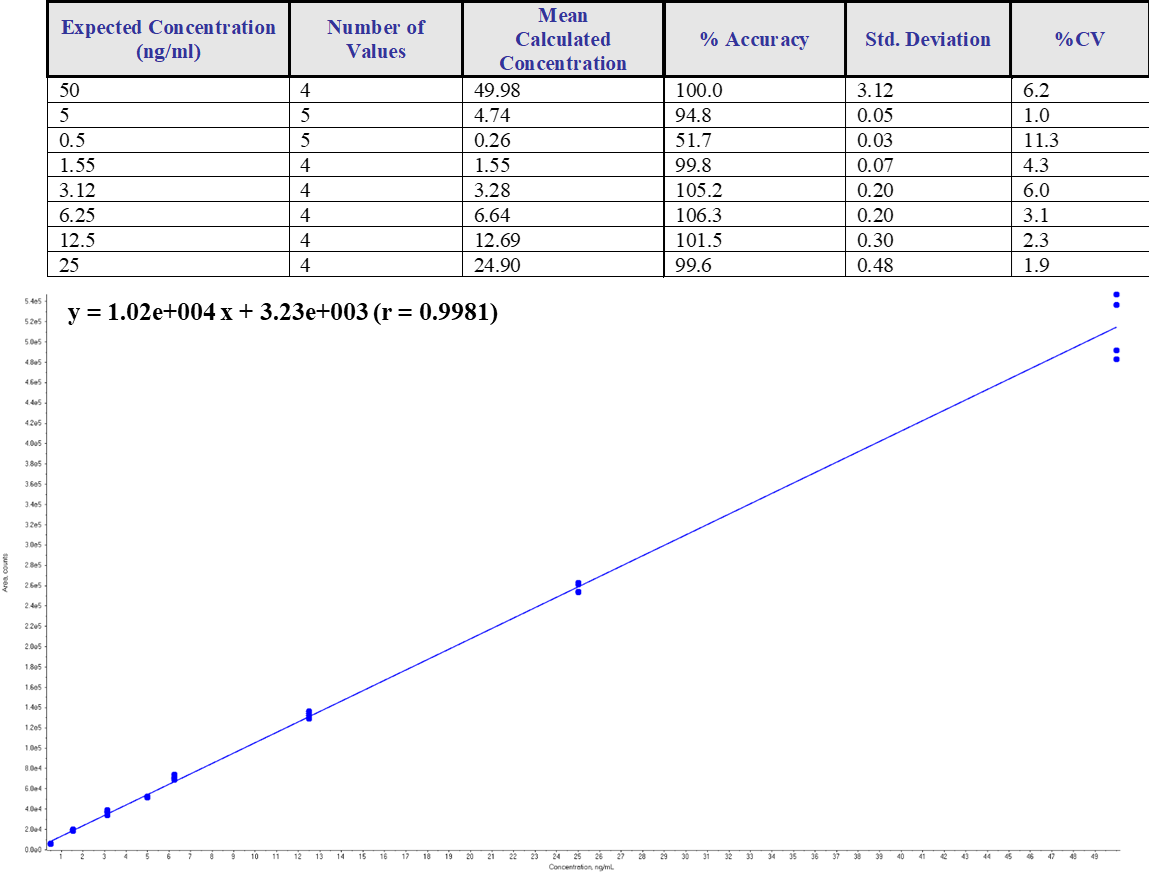


**Fig S1** : Calibration curve for 283→219 transition. Solutions of ART in methanol


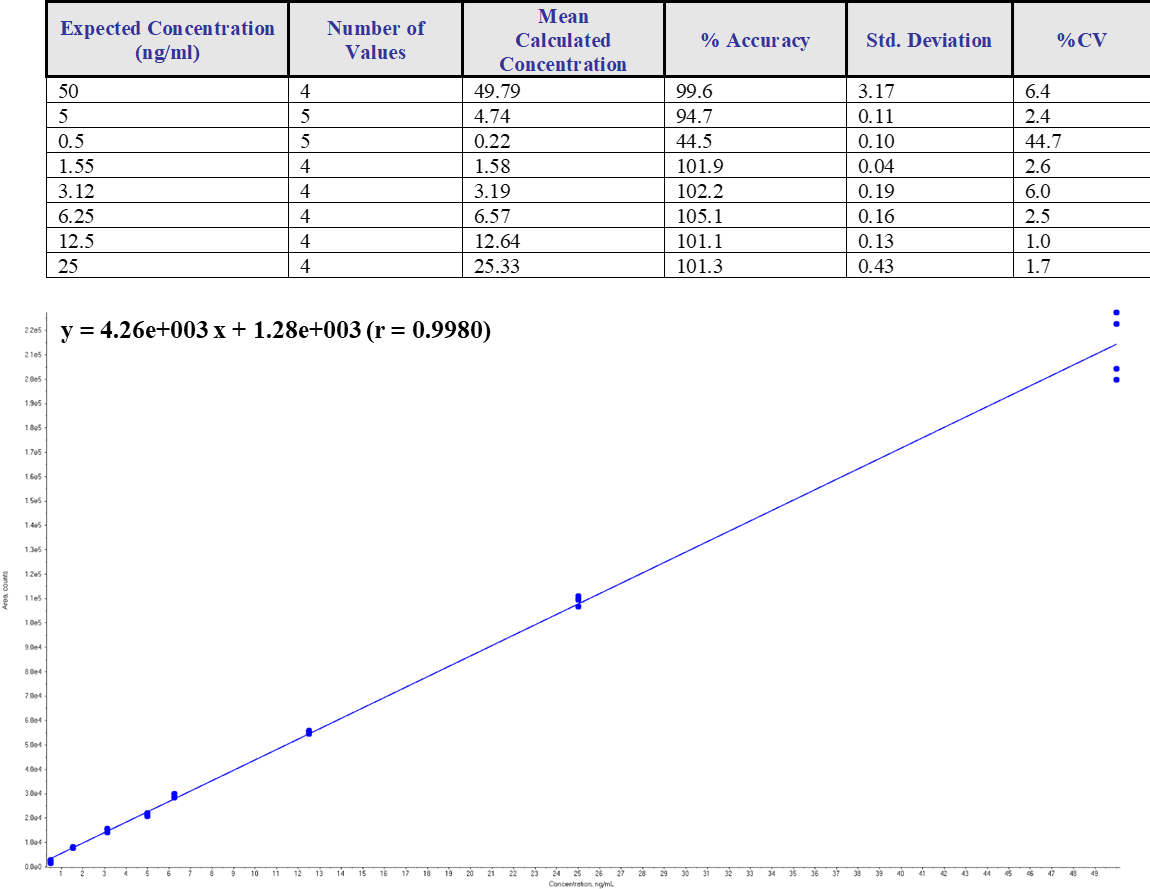


**Fig S2**: Calibration curve for 283→229 transition. Solutions of ART in methanol

**
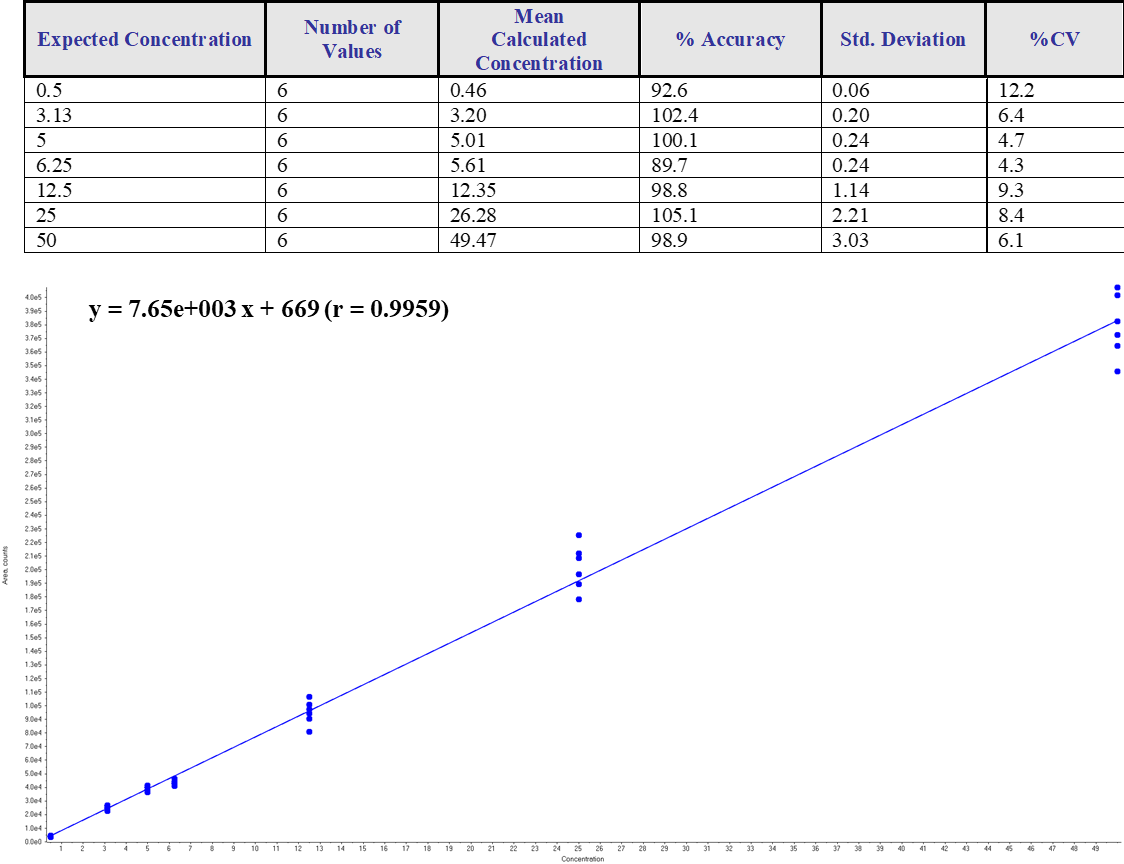
**

**Fig S3**: Calibration curve for 283→219 transition. Solutions of ART in *A. afra* tea diluted 1000 times.


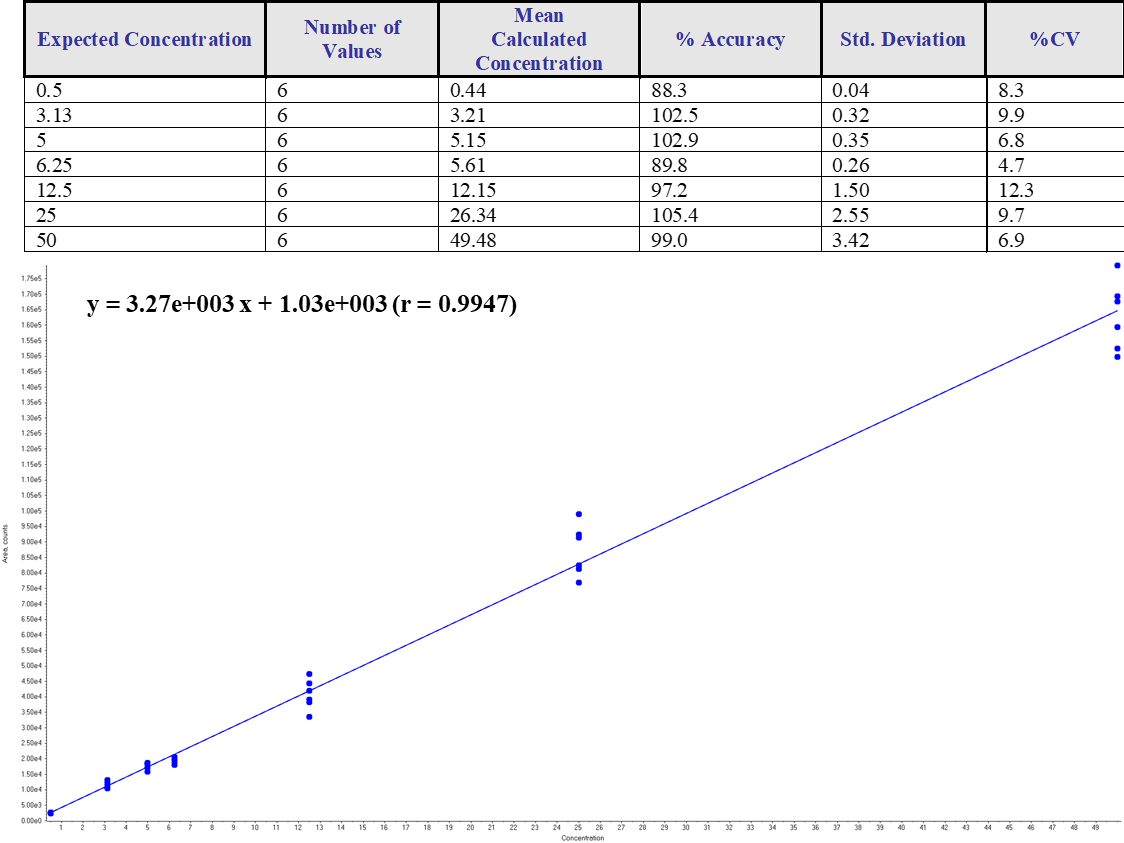


**Fig S4**: Calibration curve for 283→229 transition. Solutions of ART in *A. afra* tea diluted 1000 times


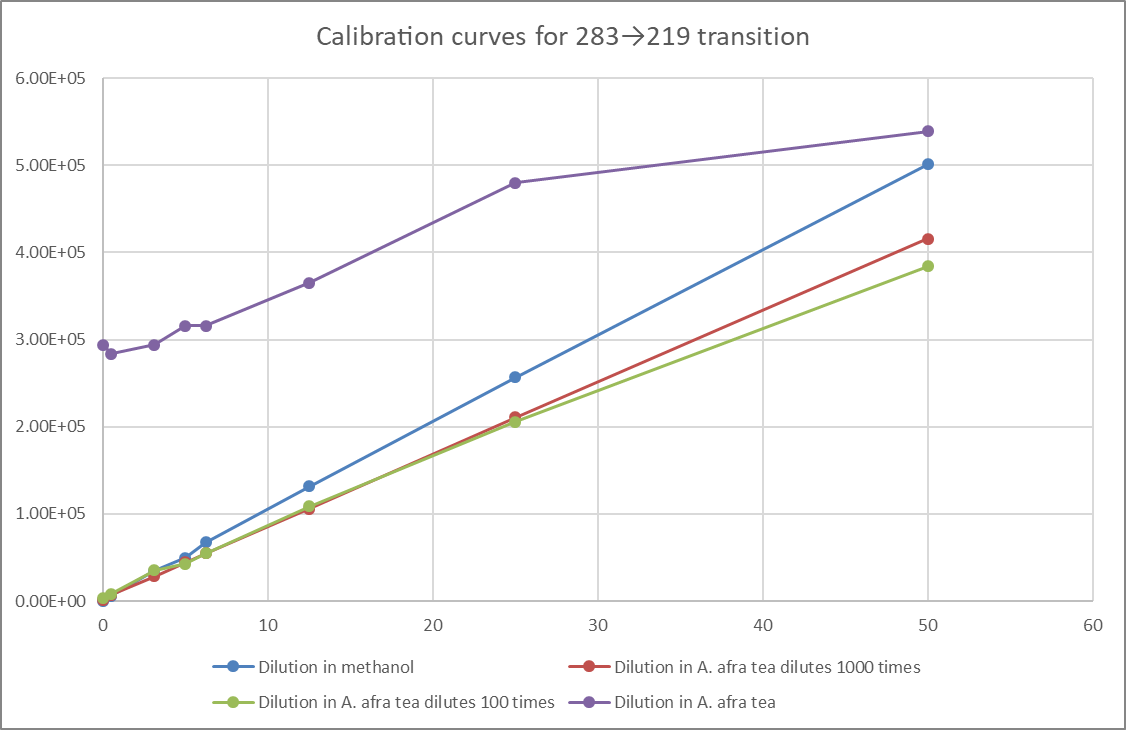


**Fig S5**: Calibration curves for 283→219 transition. Comparison between dilution in methanol, in *A. Afra* tea, in *A. afra* tea diluted 100 times, in *A. afra* tea diluted 1000 times.


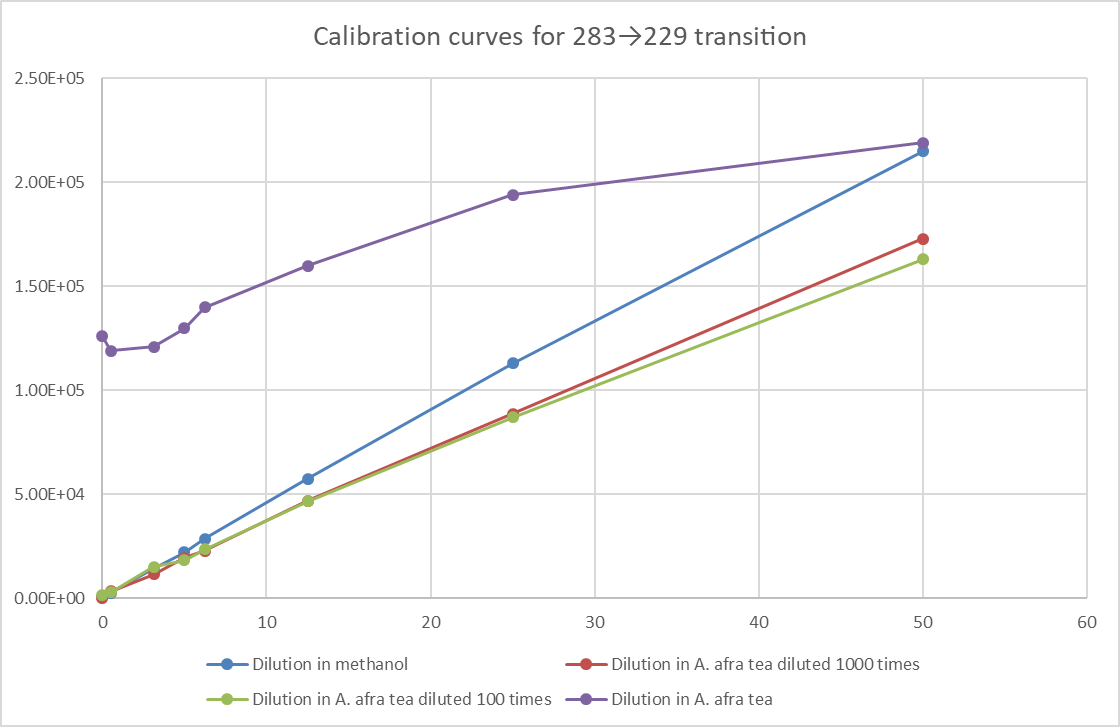


**Fig S6:** Calibration curves for 283→229 transition. Comparison between dilution in methanol, in *A. Afra* tea, in *A. afra* tea diluted 100 times, in *A. afra* tea diluted 1000 times.


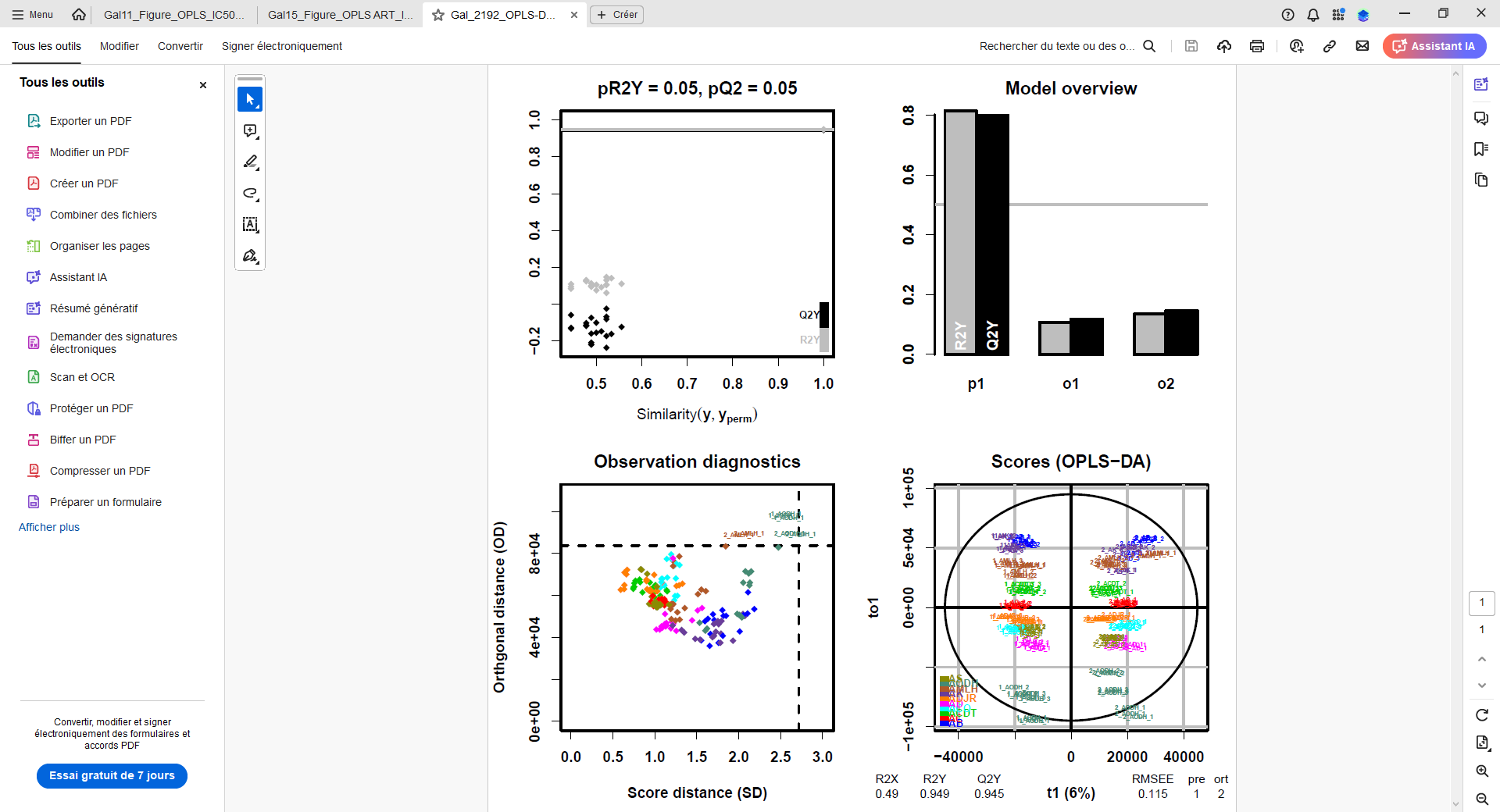


**Fig S7**: Result of the OPLS-DA analysis discriminating series 1 and series 2


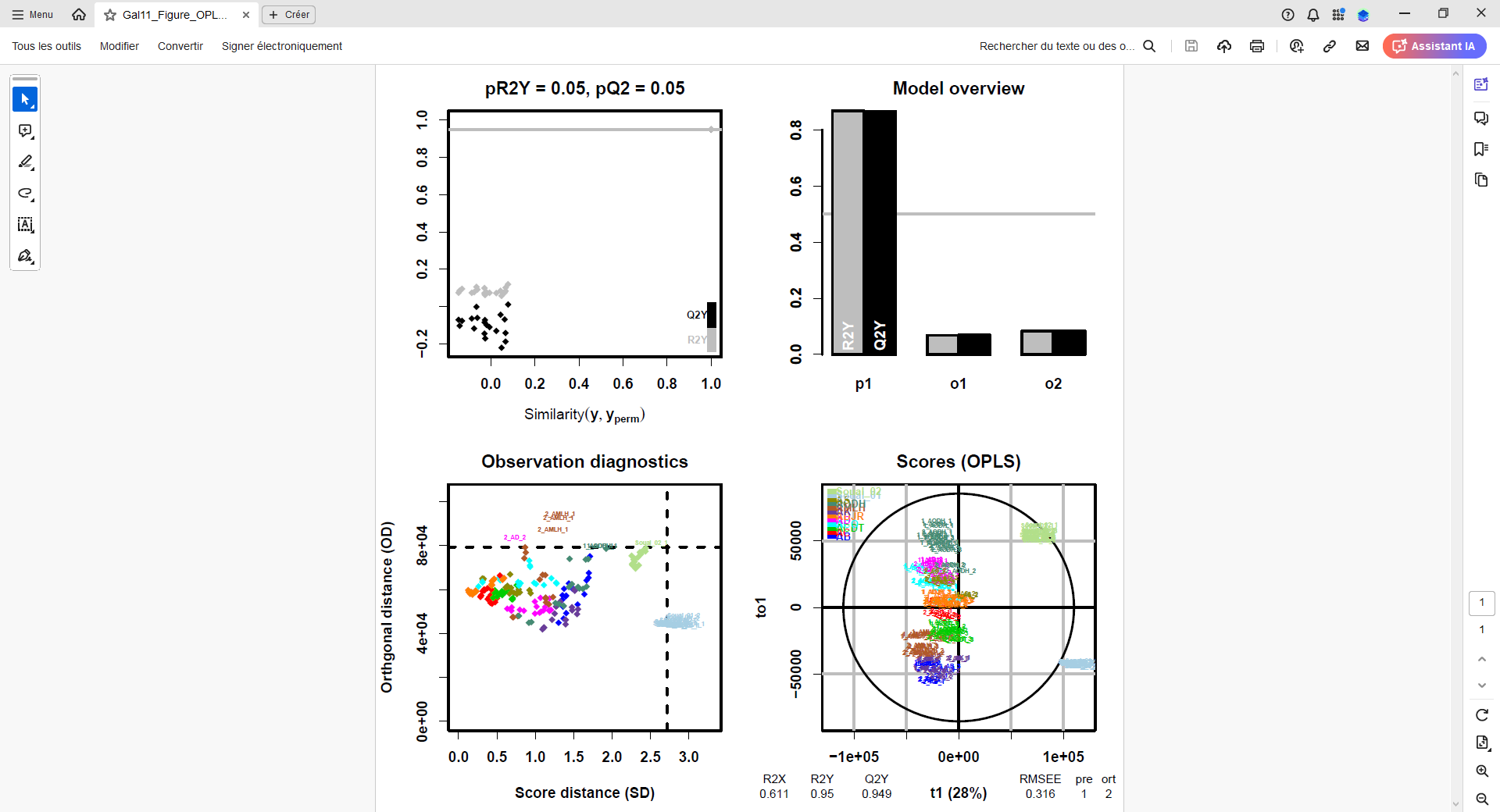


**Figure S8**: Result of the OPLS analysis with IC_50_ value as Y input


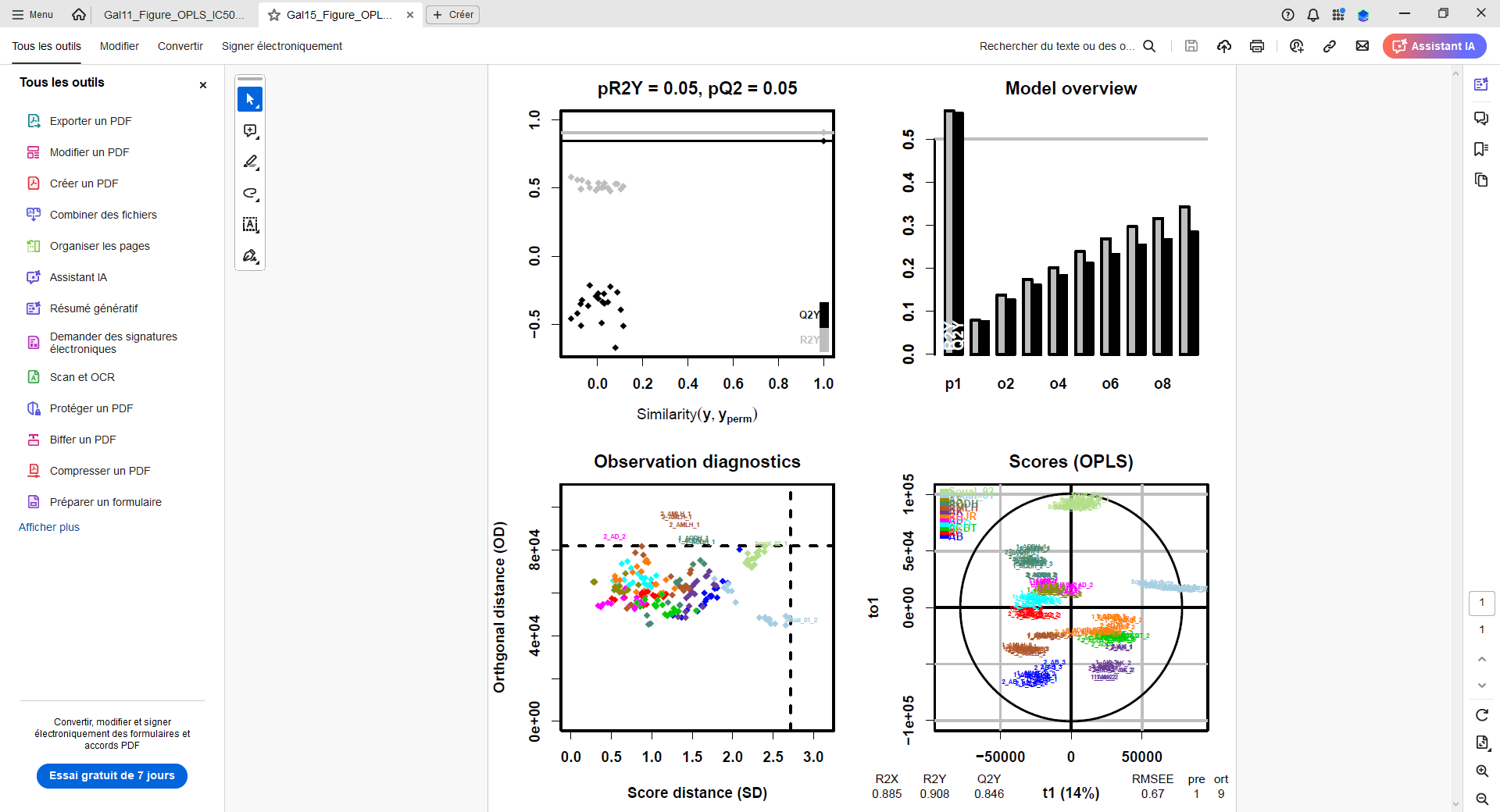


**Figure S9**: Result of the OPLS analysis with ART(tea)_IC_50_ value as Y input


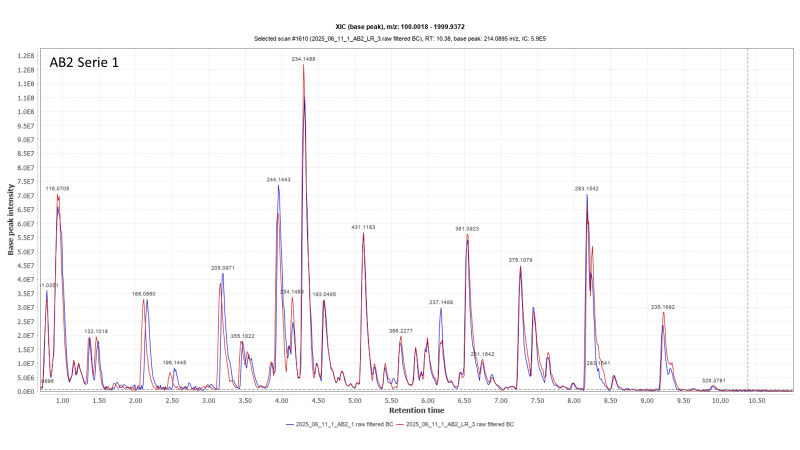


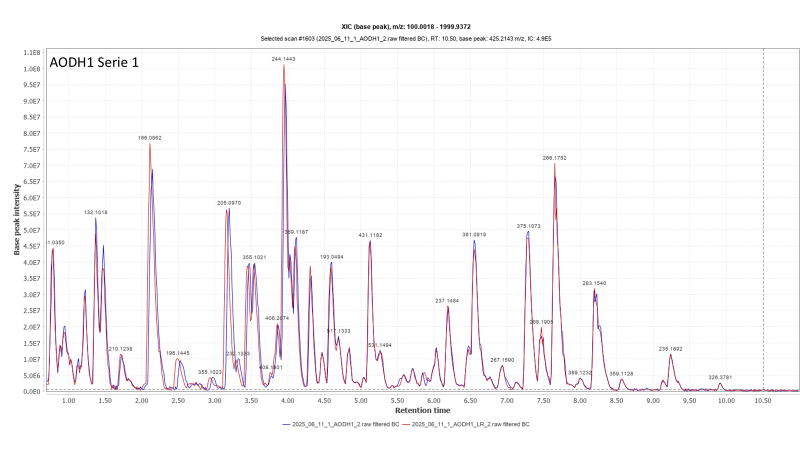


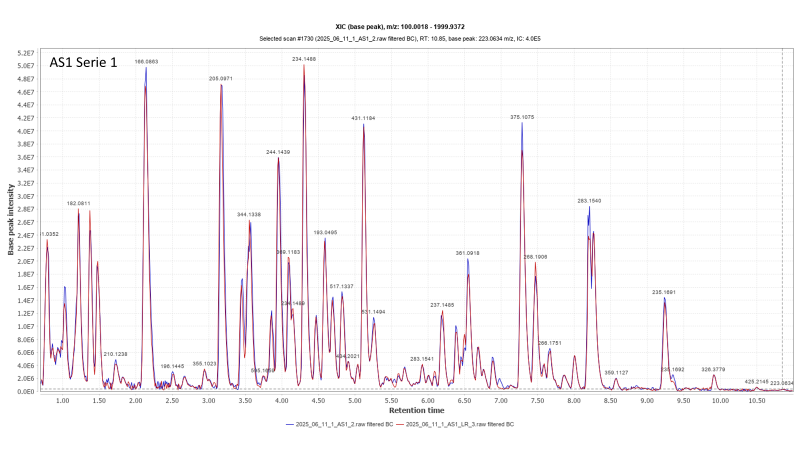


**Figure S10**: LC-HRMS chromatograms of lyophilized tea dissolved in water (in red) and frozen tea (in blue) for 3 samples of series 1 (AB2, AODH1, AS1)


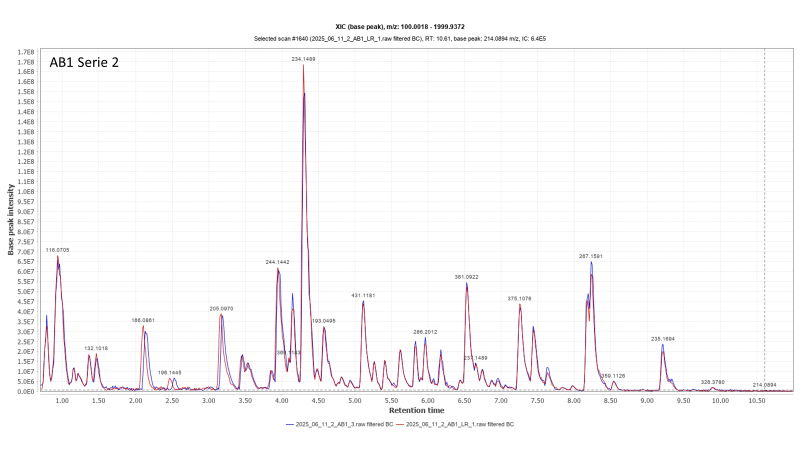


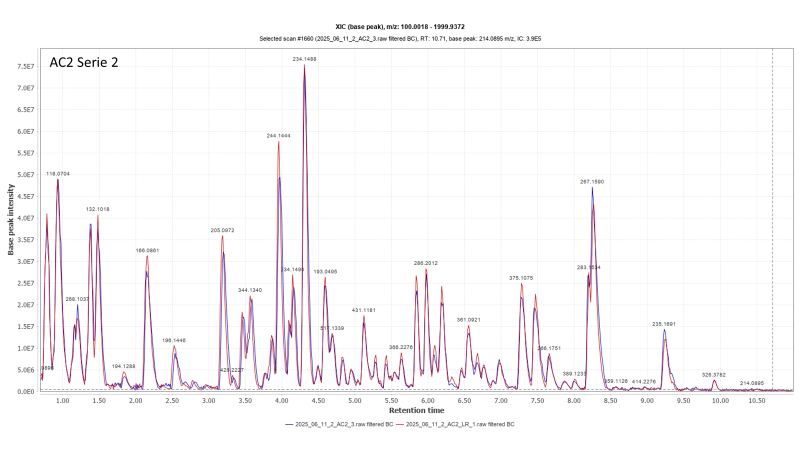


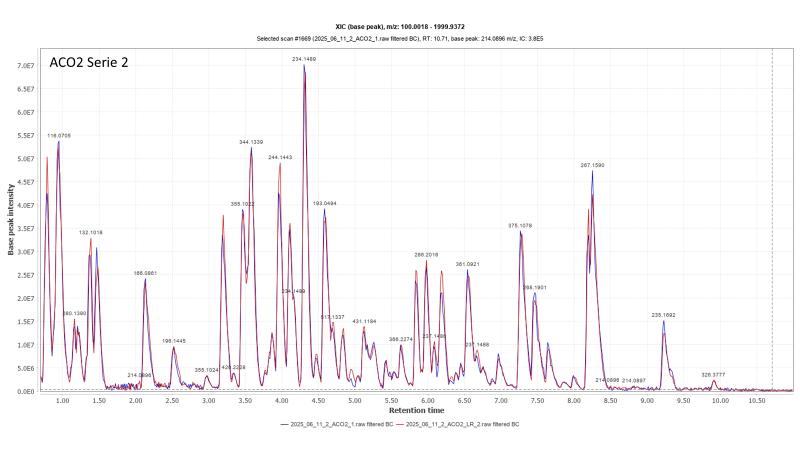


**Figure S11**: LC-HRMS chromatograms of lyophilized tea dissolved in water (in red) and frozen tea (in blue) for 3 samples of series 2 (AB1, AC2, ACO2)


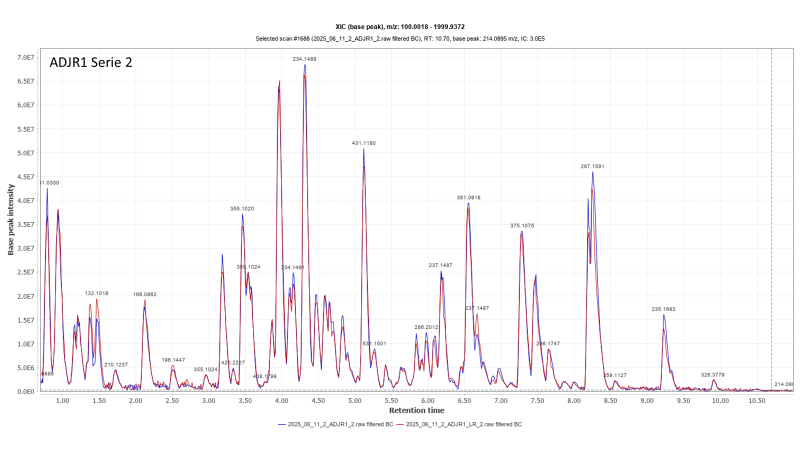

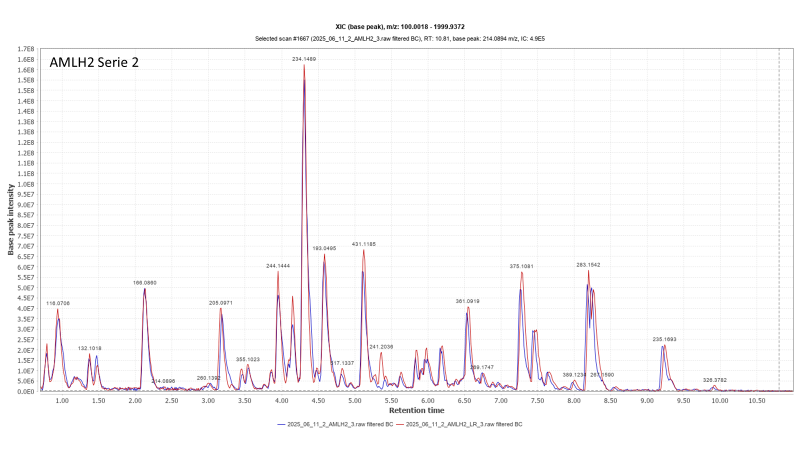


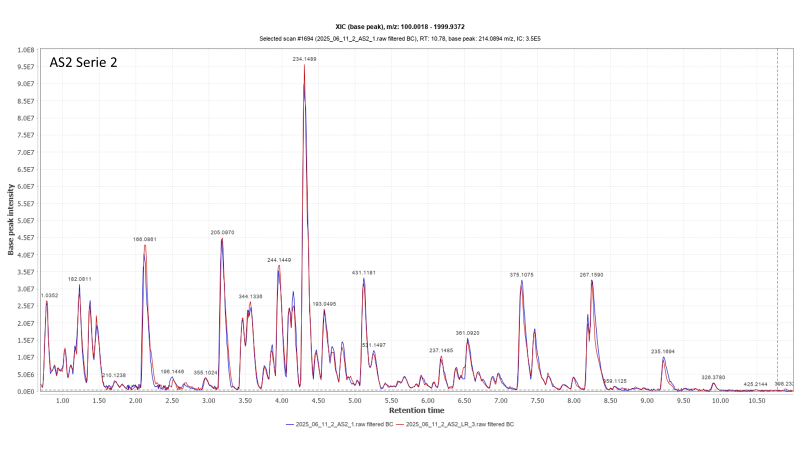


**Figure S12**: LC-HRMS chromatograms of lyophilized tea dissolved in water (in red) and frozen tea (in blue) for 3 samples of series 2 (ADJR1, AMLH2, As2)
