# Supplementary material for: Comparison of the composition and antiplasmodial activity of Artemisia annua teas using an untargeted metabolomic approach
Source: PLoS One. 2025 Aug 22;20(8):e0330682. doi: 10.1371/journal.pone.0330682 (PMC12373170; doi:10.1371/journal.pone.0330682)
Supplement: S2 File — Table S1 Location and date of sample collection, date of herbal tea preparation. Table S2 Comparison between results obtained with standard addition method, and with the use of a calibration curve made in A. afra tea diluted 1000 times, for three samples of A. annua tea (Soual_01_1, Soual_01_2, Soual_01_3) diluted 100 times. Table S3 Intraday variation for two standard solutions (5 and 12.5 ng/mL of ART in A. afra tea diluted 1000 times). Table S4 Interday variation for the standard solutions (0.5, 3.125, 5, 6.25, 12.5 ng/mL of ART in A. afra tea diluted 1000 times). Table S5 Results of determination of C_ART in A. annua teas using the 283 → 219 transition. Table S6 Results of determination of C_ART in A. annua teas using the 283 → 229 transition. Table S7 Comparison of the results for both transitions for the determination of C_ART in the teas expressed in mg/mL. Table S8: Summary of the results obtained for the 50% inhibitory concentration of the teas on 3D7 strain of P. falciparum (IC50), the concentration of ART in the teas (C_ART), the total concentration of the teas (C_Tea) and the concentration of ART corresponding to the IC50 of the teas [ART(tea)_IC50]. (DOCX) [file pone.0330682.s002.docx]

**Table S1.** Location and date of sample collection, date of herbal tea preparation.

| **Samples** | **Location** | **Departement** | **Country** | **Date of collection** | **Date of tea preparation** | |
| --- | --- | --- | --- | --- | --- | --- |
|  |  |  |  |  | **Series 1** | **Series 2** |
| ADJR_1 | Adjarra | Oueme | Benin | 27/09/2020 | 03/02/2021 | 28/02/2022 |
| ADJR_2 |  |  |  |  | 15/02/2021 | 22/02/2022 |
| ADJR_3 |  |  |  |  | 22/02/2021 | 07/02/2022 |
| ACDT_1 | Dogbo, Tota | Couffo | Benin | 13/10/2020 | 03/02/2021 | 28/02/2022 |
| ACDT_2 |  |  |  |  | 15/02/2021 | 31/01/2022 |
| ACDT_3 |  |  |  |  | 22/02/2021 | 22/02/2022 |
| AODH_1 | Ouidah | Atlantique | Benin | 15/10/2020 | 15/02/2021 | 22/02/2022 |
| AODH_2 |  |  |  |  | 03/02/2021 | 07/02/2022 |
| AODH_3 |  |  |  |  | 22/02/2021 | 01/03/2022 |
| AB_1 | Banigbe | Plateau | Benin | 19/09/2020 | 03/02/2021 | 01/03/2022 |
| AB_2 |  |  |  |  | 22/02/2021 | 31/01/2022 |
| AB_3 |  |  |  |  | 15/02/2021 | 22/02/2022 |
| ACO_1 | Cotonou | Litorral | Benin | 29/09/2020 | 03/02/2021 | 21/02/2022 |
| ACO_2 |  |  |  |  | 15/02/2021 | 28/02/2022 |
| ACO_3 |  |  |  |  | 22/02/2021 | 31/01/2022 |
| AS_1 | Seme-Kpodji | Oueme | Benin | 26/09/2020 | 22/02/2021 | 31/01/2022 |
| AS_2 |  |  |  |  | 03/02/2021 | 01/03/2022 |
| AS_3 |  |  |  |  | 15/02/2021 | 22/02/2022 |
| AD_1 | Djidja | Zou | Benin | 20/09/2020 | 15/02/2021 | 07/02/2022 |
| AD_2 |  |  |  |  | 22/02/2021 | 21/02/2022 |
| AD_3 |  |  |  |  | 03/02/2021 | 01/03/2022 |
| AC_1 | Calavi | Atlantique | Benin | 29/09/2020 | 15/02/2021 | 07/02/2022 |
| AC_2 |  |  |  |  | 22/02/2021 | 28/02/2022 |
| AC_3 |  |  |  |  | 03/02/2021 | 21/02/2022 |
| AK_1 | Ketou | PLateau | Benin | 27/09/2020 | 22/02/2021 | 21/02/2022 |
| AK_2 |  |  |  |  | 03/02/2021 | 01/03/2022 |
| AK_3 |  |  |  |  | 15/02/2021 | 07/02/2022 |
| AMLH_1 | Lokossa-Ouèdèmè | Mono | Benin | 11/10/2020 | 22/02/2021 | 31/01/2022 |
| AMLH_2 |  |  |  |  | 03/02/2021 | 28/02/2022 |
| AMLH_3 |  |  |  |  | 15/02/2021 | 21/02/2022 |
| Soual_01_1 | Soual | Tarn | France | 18/01/2021 | 19/03/2021 |  |
| Soual_01_2 |  |  |  |  | 20/03/2021 |  |
| Soual_01_3 |  |  |  |  | 21/03/2021 |  |
| Soual_02_1 |  |  |  | 12/05/2021 | 28/10/2021 |  |
| Soual_02_2 |  |  |  |  | 29/10/2021 |  |
| Soual_02_3 |  |  |  |  | 03/11/2021 |  |

**Table S2.**  Comparison between results obtained with standard addition method, and with the use of a calibration curve made in *A. afra* tea diluted 1000 times, for three samples *A. annua* of tea (Soual_01_1, Soual_01_2, Soual_01_3) diluted 100 times.

|  | **283 →219** | | |
| --- | --- | --- | --- |
| Sample Name | Calculated concentration Calibration curve AA (ng/ml) | Calculated concentration Standard addition (ng/ml) | difference % |
| Soual_01_3 100x | 5.1 | 4.7 | -7.84 |
| Soual_01_2 100x | 5.48 | 5.43 | -0.91 |
| Soual_01_1 100x | 8.15 | 8.024 | -1.55 |
|  | **283 → 229** | | |
| Soual_01_3 100x | 5.26 | 5.3 | 0.76 |
| Soual_01_2 100x | 5.45 | 5.66 | 3.85 |
| Soual_01_1 100x | 8.02 | 7.928 | -1.15 |

**Table S3**. Intraday variation for two standard solutions (5 and 12.5 ng/ml of artemisinin in *A. afra* tea diluted 1000 times).

| **283→219** | | | | |
| --- | --- | --- | --- | --- |
| **Nominal concentration (ng/ml)** | **Time of injection** | **Calculated concentration (ng/ml)** | **RE (%)^a^** | **RSD (%)^b^** |
| **5** | 17:53 | 5.44 | **3.33** | **4.80** |
|  | 21:35:00 | 5.43 |  |  |
|  | 0:30 | 5.12 |  |  |
|  | 3:40 | 5.22 |  |  |
|  | 5:32 | 4.82 |  |  |
|  | 7:07 | 4.97 |  |  |
| **12.5** | 18:25 | 13.70 | **5.73** | **4.18** |
|  | 21:51 | 13.60 |  |  |
|  | 0:46 | 13.40 |  |  |
|  | 4:12 | 13.40 |  |  |
|  | 5:48 | 13.00 |  |  |
|  | 7:23 | 12.20 |  |  |
|  |  |  |  |  |
| **283→229** | | | | |
| **Nominal concentration (ng/ml)** | **Time of injection** | **Calculated concentration (ng/ml)** | **RE (%)** | **RSD (RSD)** |
| **5** | 17:53 | 5.86 | **5.47** | **6.76** |
|  | 21:35:00 | 5.46 |  |  |
|  | 0:30 | 5.02 |  |  |
|  | 3:40 | 5.29 |  |  |
|  | 5:32 | 4.85 |  |  |
|  | 7:07 | 5.16 |  |  |
| **12.5** | 18:25 | 14 | **7.60** | **4.30** |
|  | 21:51 | 13.6 |  |  |
|  | 0:46 | 13.8 |  |  |
|  | 4:12 | 13.7 |  |  |
|  | 5:48 | 13.2 |  |  |
|  | 7:23 | 12.4 |  |  |

1. RSD(%) = (Standard deviation/Mean)x100.
2. RE(%) = [(Calculated concentration -Nominal concentration)/Nominal concentration]x100

**Table S4**. Interday variation for the standard solutions (0.5, 3.125, 5, 6.25, 12.5 ng/ml of ART in *A. afra* tea diluted 1000 times)

| **Nominal concentration** | **Calculated concentration 283→219** | | | **Mean** | **SD** | **RE(%)^a^** | **RSD(%)^b^** |
| --- | --- | --- | --- | --- | --- | --- | --- |
|  | **Day 1** | **Day2** | **Day3** |  |  |  |  |
| 0.5 | 0.468 | 0.526 | 0.531 | 0.51 | 0.04 | 1.67 | 6.89 |
| 3.12 | 3.14 | 3.38 | 3.26 | 3.26 | 0.12 | 4.49 | 3.68 |
| 5 | 4.65 | 4.89 | 5.16 | 4.90 | 0.26 | -2.00 | 5.21 |
| 6.25 | 5.54 | 5.99 | 5.65 | 5.73 | 0.23 | -8.37 | 4.10 |
| 12.5 | 10.5 | 11.8 | 12.7 | 11.67 | 1.11 | -6.67 | 9.48 |
| 25 | 23.2 | 24.7 | 27.2 | 25.03 | 2.02 | 0.13 | 8.07 |
| 50 | 45.1 | 48.6 | 49.9 | 47.87 | 2.48 | -4.27 | 5.19 |
|  | **Calculated concentration 283→229** | | |  |  |  |  |
|  | **Day 1** | **Day2** | **Day3** |  |  |  |  |
| 0.5 | 0.44 | 0.392 | 0.448 | 0.43 | 0.03 | -14.67 | 7.10 |
| 3.12 | 2.88 | 3.43 | 3.22 | 3.18 | 0.28 | 1.82 | 8.74 |
| 5 | 4.53 | 4.94 | 5.27 | 4.91 | 0.37 | -1.73 | 7.55 |
| 6.25 | 5.67 | 5.96 | 5.75 | 5.79 | 0.15 | -7.31 | 2.59 |
| 12.5 | 9.93 | 11.4 | 12.5 | 11.28 | 1.29 | -9.79 | 11.43 |
| 25 | 23.2 | 24.5 | 27.6 | 25.10 | 2.26 | 0.40 | 9.01 |
| 50 | 45.4 | 48.4 | 50.9 | 48.23 | 2.75 | -3.53 | 5.71 |

1. RSD(%) = (Standard deviation/Mean)x100.
2. RE(%) = [(Calculated concentration -Nominal concentration)/Nominal concentration]x100

**Table S5:** Results of determinantion of C_ART in *A. annua* teas using the 283→219 transition

| **Transition 283→219** | | | | | | | |
| --- | --- | --- | --- | --- | --- | --- | --- |
| **Samples** | **Series** | **Dilution** | **Nb of independent analysis** | **Mean measured concentration (ng/ml)** | **SD** | **RSD (%)** | **C_ART in tea (mg/l)** |
| AB_1 | 1 | 1000 | 3 | 13.79 | 1.86 | 13.47 | 13.79 |
| AB_1 | 2 | 1000 | 3 | 10.31 | 0.77 | 7.48 | 10.31 |
| AB_2 | 1 | 1000 | 3 | 13.03 | 0.68 | 5.22 | 13.03 |
| AB_2 | 2 | 1000 | 3 | 9.69 | 1.05 | 10.85 | 9.69 |
| AB_3 | 1 | 1000 | 3 | 12.96 | 1.16 | 8.99 | 12.96 |
| AB_3 | 2 | 1000 | 3 | 6.55 | 0.21 | 3.13 | 6.55 |
| AC_1 | 1 | 1000 | 1 | 6.58 |  |  | 6.58 |
| AC_1 | 2 | 1000 | 1 | 6.61 |  |  | 6.61 |
| AC_2 | 1 | 1000 | 1 | 5.74 |  |  | 5.74 |
| AC_2 | 2 | 1000 | 1 | 4.74 |  |  | 4.74 |
| AC_3 | 1 | 1000 | 1 | 4.85 |  |  | 4.85 |
| AC_3 | 2 | 100 | 1 | 42.00 |  |  | 4.20 |
| ACDT_1 | 1 | 1000 | 1 | 9.54 |  |  | 9.54 |
| ACDT_1 | 2 | 1000 | 1 | 6.27 |  |  | 6.27 |
| ACDT_2 | 1 | 1000 | 1 | 8.46 |  |  | 8.46 |
| ACDT_2 | 2 | 1000 | 1 | 6.91 |  |  | 6.91 |
| ACDT_3 | 1 | 1000 | 1 | 7.52 |  |  | 7.52 |
| ACDT_3 | 2 | 1000 | 1 | 6.12 |  |  | 6.12 |
| ACO_1 | 1 | 1000 | 1 | 4.33 |  |  | 4.33 |
| ACO_1 | 2 | 100 | 1 | 25.9 |  |  | 2.59 |
| ACO_2 | 1 | 1000 | 1 | 6.28 |  |  | 6.28 |
| ACO_2 | 2 | 1000 | 1 | 5.82 |  |  | 5.82 |
| ACO_3 | 1 | 1000 | 1 | 9 |  |  | 9 |
| ACO_3 | 2 | 1000 | 1 | 6.7 |  |  | 6.7 |
| AD_1 | 1 | 1000 | 1 | 3.41 |  |  | 3.41 |
| AD_1 | 2 | 1000 | 1 | 3.62 |  |  | 3.62 |
| AD_2 | 1 | 1000 | 3 | 3.85 | 0.27 | 6.89 | 3.85 |
| AD_2 | 2 | 100 | 1 | 37 |  |  | 3.7 |
| AD_3 | 1 | 1000 | 3 | 4.75 | 0.57 | 12.04 | 4.75 |
| AD_3 | 2 | 100 | 1 | 29.6 |  |  | 2.96 |
| ADJR_1 | 1 | 1000 | 1 | 4.32 |  |  | 4.32 |
| ADJR_1 | 2 | 1000 | 1 | 6.31 |  |  | 6.31 |
| ADJR_2 | 1 | 1000 | 1 | 4.54 |  |  | 4.54 |
| ADJR_2 | 2 | 1000 | 1 | 4.57 |  |  | 4.57 |
| ADJR_3 | 1 | 1000 | 1 | 8.02 |  |  | 8.02 |
| ADJR_3 | 2 | 1000 | 1 | 7.91 |  |  | 7.91 |
| AK_1 | 1 | 1000 | 4 | 15.55 | 1.98 | 12.74 | 15.55 |
| AK_1 | 2 | 1000 | 4 | 4.20 | 0.58 | 13.77 | 4.20 |
| AK_2 | 1 | 1000 | 4 | 15.71 | 1.35 | 8.56 | 15.71 |
| AK_2 | 2 | 1000 | 2 | 7.88 | 0.26 | 3.32 | 7.88 |
| AK_3 | 1 | 1000 | 3 | 11.59 | 0.89 | 7.71 | 11.59 |
| AK_3 | 2 | 1000 | 3 | 10.21 | 0.89 | 8.67 | 10.21 |
| AMLH_1 | 1 | 1000 | 1 | 8.01 |  |  | 8.01 |
| AMLH_1 | 2 | 1000 | 1 | 7.57 |  |  | 7.57 |
| AMLH_2 | 1 | 1000 | 1 | 8.22 |  |  | 8.22 |
| AMLH_2 | 2 | 1000 | 1 | 10.5 |  |  | 10.5 |
| AMLH_3 | 1 | 1000 | 1 | 12.6 |  |  | 12.6 |
| AMLH_3 | 2 | 1000 | 1 | 9.27 |  |  | 9.27 |
| AODH_1 | 1 | 1000 | 1 | 5.24 |  |  | 5.24 |
| AODH_1 | 2 | 1000 | 1 | 3.85 |  |  | 3.85 |
| AODH_2 | 1 | 1000 | 1 | 5.68 |  |  | 5.68 |
| AODH_2 | 2 | 1000 | 1 | 3.29 |  |  | 3.29 |
| AODH_3 | 1 | 1000 | 3 | 3.54 | 0.26 | 7.33 | 3.54 |
| AODH_3 | 2 | 100 | 1 | 19.00 |  |  | 1.90 |
| AS_1 | 1 | 1000 | 1 | 4.26 |  |  | 4.26 |
| AS_1 | 2 | 1000 | 1 | 3.51 |  |  | 3.51 |
| AS_2 | 1 | 100 | 1 | 25.70 |  |  | 2.57 |
| AS_2 | 2 | 100 | 1 | 31.5 |  |  | 3.15 |
| AS_3 | 1 | 1000 | 1 | 4 |  |  | 4 |
| AS_3 | 2 | 100 | 1 | 20.9 |  |  | 2.09 |
| Soual_01_1 | - | 100 | 3 | 7.55 | 0.94 | 12.40 | 0.75 |
| Soual_01_2 | - | 100 | 4 | 5.57 | 0.46 | 8.18 | 0.56 |
| Soual_01_3 | - | 100 | 3 | 4.93 | 0.21 | 4.22 | 0.49 |
| Soual_02_1 | - | 100 | 1 | 18.2 |  |  | 1.82 |
| Soual_02_2 | - | 100 | 1 | 18.8 |  |  | 1.88 |
| Soual_02_3 | - | 100 | 1 | 17.9 |  |  | 1.79 |

**Table S6:** Results of determination of C_ART in *A. annua* teas using the 283→229 transition

| **Transition 283→229** | | | | | | | |
| --- | --- | --- | --- | --- | --- | --- | --- |
| **Samples** | **Series** | **Dilution** | **Nb of independent analysis** | **Mean measured concentration (ng/ml)** | **SD** | **RSD (%)** | **C_ART in tea (mg/l)** |
| AB_1 | 1 | 1000 | 3 | 13.53 | 1.70 | 12.59 | 13.53 |
| AB_1 | 2 | 1000 | 3 | 10.65 | 1.19 | 11.22 | 10.65 |
| AB_2 | 1 | 1000 | 3 | 12.90 | 0.72 | 5.59 | 12.90 |
| AB_2 | 2 | 1000 | 3 | 9.63 | 1.31 | 13.62 | 9.63 |
| AB_3 | 1 | 1000 | 3 | 13.03 | 1.13 | 8.70 | 13.03 |
| AB_3 | 2 | 1000 | 3 | 6.67 | 0.32 | 4.85 | 6.67 |
| AC_1 | 1 | 1000 | 1 | 6.64 |  |  | 6.64 |
| AC_1 | 2 | 1000 | 1 | 7.2 |  |  | 7.2 |
| AC_2 | 1 | 1000 | 1 | 5.75 |  |  | 5.75 |
| AC_2 | 2 | 1000 | 1 | 5.06 |  |  | 5.06 |
| AC_3 | 1 | 1000 | 1 | 4.5 |  |  | 4.5 |
| AC_3 | 2 | 100 | 1 | 41.1 |  |  | 4.11 |
| ACDT_1 | 1 | 1000 | 1 | 9.95 |  |  | 9.95 |
| ACDT_1 | 2 | 1000 | 1 | 6.33 |  |  | 6.33 |
| ACDT_2 | 1 | 1000 | 1 | 8.8 |  |  | 8.8 |
| ACDT_2 | 2 | 1000 | 1 | 7.27 |  |  | 7.27 |
| ACDT_3 | 1 | 1000 | 1 | 6.63 |  |  | 6.63 |
| ACDT_3 | 2 | 1000 | 1 | 6.25 |  |  | 6.25 |
| ACO_1 | 1 | 1000 | 1 | 4.58 |  |  | 4.58 |
| ACO_1 | 2 | 100 | 1 | 24.6 |  |  | 2.46 |
| ACO_2 | 1 | 1000 | 1 | 6.64 |  |  | 6.64 |
| ACO_2 | 2 | 1000 | 1 | 6.28 |  |  | 6.28 |
| ACO_3 | 1 | 1000 | 1 | 8.95 |  |  | 8.95 |
| ACO_3 | 2 | 1000 | 1 | 6.89 |  |  | 6.89 |
| AD_1 | 1 | 1000 | 1 | 3.27 |  |  | 3.41 |
| AD_1 | 2 | 1000 | 1 | 3.71 |  |  | 3.71 |
| AD_2 | 1 | 1000 | 3 | 3.98 | 0.26 | 6.53 | 3.98 |
| AD_2 | 2 | 100 | 1 | 35.8 |  |  | 3.58 |
| AD_3 | 1 | 1000 | 3 | 4.77 | 0.72 | 15.06 | 4.77 |
| AD_3 | 2 | 100 | 1 | 29.6 |  |  | 2.96 |
| ADJR_1 | 1 | 1000 | 1 | 4.2 |  |  | 4.2 |
| ADJR_1 | 2 | 1000 | 1 | 6.42 |  |  | 6.42 |
| ADJR_2 | 1 | 1000 | 1 | 4.66 |  |  | 4.66 |
| ADJR_2 | 2 | 1000 | 1 | 4.47 |  |  | 4.47 |
| ADJR_3 | 1 | 1000 | 1 | 8.44 |  |  | 8.44 |
| ADJR_3 | 2 | 1000 | 1 | 8.1 |  |  | 8.1 |
| AK_1 | 1 | 1000 | 4 | 14.78 | 2.34 | 15.86 | 14.78 |
| AK_1 | 2 | 1000 | 4 | 4.31 | 0.39 | 9.12 | 4.31 |
| AK_2 | 1 | 1000 | 4 | 15.75 | 1.13 | 7.16 | 15.75 |
| AK_2 | 2 | 1000 | 2 | 8.13 | 0.52 | 6.35 | 8.13 |
| AK_3 | 1 | 1000 | 3 | 11.47 | 1.32 | 11.51 | 11.47 |
| AK_3 | 2 | 1000 | 3 | 10.31 | 1.06 | 10.27 | 10.31 |
| AMLH_1 | 1 | 1000 | 1 | 7.6 |  |  | 7.6 |
| AMLH_1 | 2 | 1000 | 1 | 7.75 |  |  | 7.75 |
| AMLH_2 | 1 | 1000 | 1 | 8.52 |  |  | 8.52 |
| AMLH_2 | 2 | 1000 | 1 | 10.5 |  |  | 10.5 |
| AMLH_3 | 1 | 1000 | 1 | 12.8 |  |  | 12.8 |
| AMLH_3 | 2 | 1000 | 1 | 10.1 |  |  | 10.1 |
| AODH_1 | 1 | 1000 | 1 | 5.64 |  |  | 5.64 |
| AODH_1 | 2 | 1000 | 1 | 3.91 |  |  | 3.91 |
| AODH_2 | 1 | 1000 | 1 | 5.79 |  |  | 5.79 |
| AODH_2 | 2 | 1000 | 1 | 3.3 |  |  | 3.3 |
| AODH_3 | 1 | 1000 | 3 | 3.46 | 0.11 | 3.06 | 3.46 |
| AODH_3 | 2 | 100 | 1 | 19.10 |  |  | 1.91 |
| AS_1 | 1 | 1000 | 1 | 4.21 |  |  | 4.21 |
| AS_1 | 2 | 1000 | 1 | 3.46 |  |  | 3.46 |
| AS_2 | 1 | 100 | 1 | 27.00 |  |  | 2.7 |
| AS_2 | 2 | 100 | 1 | 31.3 |  |  | 3.13 |
| AS_3 | 1 | 1000 | 1 | 4.1 |  |  | 4.1 |
| AS_3 | 2 | 100 | 1 | 21.5 |  |  | 2.15 |
| Soual_01_1 | - | 100 | 3 | 7.36 | 1.07 | 14.56 | 0.74 |
| Soual_01_2 | - | 100 | 4 | 5.46 | 0.57 | 10.41 | 0.55 |
| Soual_01_3 | - | 100 | 3 | 4.95 | 0.57 | 11.43 | 0.50 |
| Soual_02_1 | - | 100 | 1 | 17.8 |  |  | 1.78 |
| Soual_02_2 | - | 100 | 1 | 18.4 |  |  | 1.84 |
| Soual_02_3 | - | 100 | 1 | 17.3 |  |  | 1.73 |

**Table S7:** Comparison of the results for both transitions for the determination of C_ART in the teas expressed in mg/ml.

| **Samples** | **Series** | **Transition 283→219** | **Transition 283→229** | **Difference (%)** |
| --- | --- | --- | --- | --- |
| AB_1 | 1 | 13.79 | 13.53 | 1.86 |
| AB_1 | 2 | 10.31 | 10.65 | -3.27 |
| AB_2 | 1 | 13.03 | 12.90 | 1.02 |
| AB_2 | 2 | 9.69 | 9.63 | 0.62 |
| AB_3 | 1 | 12.96 | 13.03 | -0.58 |
| AB_3 | 2 | 6.55 | 6.67 | -1.73 |
| AC_1 | 1 | 6.58 | 6.64 | -0.91 |
| AC_1 | 2 | 6.61 | 7.20 | -8.93 |
| AC_2 | 1 | 5.74 | 5.75 | -0.17 |
| AC_2 | 2 | 4.74 | 5.06 | -6.75 |
| AC_3 | 1 | 4.85 | 4.50 | 7.22 |
| AC_3 | 2 | 4.20 | 4.11 | 2.14 |
| ACDT_1 | 1 | 9.54 | 9.95 | -4.30 |
| ACDT_1 | 2 | 6.27 | 6.33 | -0.96 |
| ACDT_2 | 1 | 8.46 | 8.80 | -4.02 |
| ACDT_2 | 2 | 6.91 | 7.27 | -5.21 |
| ACDT_3 | 1 | 7.52 | 6.63 | 11.84 |
| ACDT_3 | 2 | 6.12 | 6.25 | -2.12 |
| ACO_1 | 1 | 4.33 | 4.58 | -5.77 |
| ACO_1 | 2 | 2.59 | 2.46 | 5.02 |
| ACO_2 | 1 | 6.28 | 6.64 | -5.73 |
| ACO_2 | 2 | 5.82 | 6.28 | -7.90 |
| ACO_3 | 1 | 9.00 | 8.95 | 0.56 |
| ACO_3 | 2 | 6.70 | 6.89 | -2.84 |
| AD_1 | 1 | 3.41 | 3.41 | 0.00 |
| AD_1 | 2 | 3.62 | 3.71 | -2.49 |
| AD_2 | 1 | 3.85 | 3.98 | -3.38 |
| AD_2 | 2 | 3.70 | 3.58 | 3.24 |
| AD_3 | 1 | 4.75 | 4.77 | -0.42 |
| AD_3 | 2 | 2.96 | 2.96 | 0.00 |
| ADJR_1 | 1 | 4.32 | 4.20 | 2.78 |
| ADJR_1 | 2 | 6.31 | 6.42 | -1.74 |
| ADJR_2 | 1 | 4.54 | 4.66 | -2.64 |
| ADJR_2 | 2 | 4.57 | 4.47 | 2.19 |
| ADJR_3 | 1 | 8.02 | 8.44 | -5.24 |
| ADJR_3 | 2 | 7.91 | 8.10 | -2.40 |
| AK_1 | 1 | 15.55 | 14.78 | 4.95 |
| AK_1 | 2 | 4.20 | 4.31 | -2.74 |
| AK_2 | 1 | 15.71 | 15.75 | -0.24 |
| AK_2 | 2 | 7.88 | 8.13 | -3.11 |
| AK_3 | 1 | 11.59 | 11.47 | 1.06 |
| AK_3 | 2 | 10.21 | 10.31 | -0.98 |
| AMLH_1 | 1 | 8.01 | 7.60 | 5.12 |
| AMLH_1 | 2 | 7.57 | 7.75 | -2.38 |
| AMLH_2 | 1 | 8.22 | 8.52 | -3.65 |
| AMLH_2 | 2 | 10.50 | 10.50 | 0.00 |
| AMLH_3 | 1 | 12.60 | 12.80 | -1.59 |
| AMLH_3 | 2 | 9.27 | 10.10 | -8.95 |
| AODH_1 | 1 | 5.24 | 5.64 | -7.63 |
| AODH_1 | 2 | 3.85 | 3.91 | -1.56 |
| AODH_2 | 1 | 5.68 | 5.79 | -1.94 |
| AODH_2 | 2 | 3.29 | 3.30 | -0.30 |
| AODH_3 | 1 | 3.54 | 3.46 | 2.26 |
| AODH_3 | 2 | 1.90 | 1.91 | -0.53 |
| AS_1 | 1 | 4.26 | 4.21 | 1.17 |
| AS_1 | 2 | 3.51 | 3.46 | 1.42 |
| AS_2 | 1 | 2.57 | 2.70 | -5.06 |
| AS_2 | 2 | 3.15 | 3.13 | 0.63 |
| AS_3 | 1 | 4.00 | 4.10 | -2.50 |
| AS_3 | 2 | 2.09 | 2.15 | -2.87 |
| Soual_01_1 | - | 0.75 | 0.74 | 1.33 |
| Soual_01_2 | - | 0.56 | 0.55 | 1.79 |
| Soual_01_3 | - | 0.49 | 0.50 | -2.04 |
| Soual_02_1 | - | 1.82 | 1.78 | 2.20 |
| Soual_02_2 | - | 1.88 | 1.84 | 2.13 |
| Soual_02_3 | - | 1.79 | 1.73 | 3.35 |

**Table S8:** Summary of the results obtained for the 50% inhibitory concentration of the teas on 3D7 strain of *P. falciparum* (IC_50_), the concentration of ART in the teas (C_ART), the total concentration of the teas (C_Tea) and the concentration of ART corresponding to the IC_50_ of the teas [ART(tea)_IC_50_]^a^

|  | **IC_50_**  **(µg/ml)** | | **C_ART**  **(mg/l)** | | **C_Tea**  **(mg/ml)** | | **ART(tea)_IC_50_**  **(ng/ml)** | |
| --- | --- | --- | --- | --- | --- | --- | --- | --- |
|  | **Series 1** | **Series 2** | **Series 1** | **Series 2** | **Series 1** | **Series 2** | **Series 1** | **Series 2** |
| **ADJR1** | 1.57 | 0.85 | 4.32 | 6.31 | 0.92 | 1.18 | 7.36 | 4.56 |
| **ADJR2** | 1.14 | 0.99 | 4.54 | 4.57 | 0.96 | 0.94 | 5.39 | 4.81 |
| **ADJR3** | 0.60 | 0.73 | 8.02 | 7.91 | 1.14 | 1.19 | 4.20 | 4.85 |
|  |  |  |  |  |  |  |  |  |
| **AS1** | 0.78 | 1.10 | 4.26 | 3.51 | 1.14 | 1.22 | 2.93 | 3.16 |
| **AS2** | 0.80 | 0.93 | 2.57 | 3.15 | 0.90 | 1.24 | 2.29 | 2.37 |
| **AS3** | 0.63 | 1.36 | 4 | 2.09 | 1.18 | 1.04 | 2.12 | 2.74 |
|  |  |  |  |  |  |  |  |  |
| **AD1** | 0.80 | 0.81 | 3.41 | 3.62 | 1.15 | 1.26 | 2.36 | 2.34 |
| **AD2** | 0.69 | 1.14 | 3.85 | 3.7 | 1.27 | 1.16 | 2.08 | 3.60 |
| **AD3** | 0.58 | 0.92 | 4.75 | 2.96 | 1.19 | 1.13 | 2.32 | 2.40 |
|  |  |  |  |  |  |  |  |  |
| **AB1** | 0.23 | 0.23 | 13.79 | 10.31 | 1.57 | 1.38 | 2.05 | 1.72 |
| **AB2** | 0.22 | 0.26 | 13.03 | 9.69 | 1.40 | 1.38 | 2.08 | 1.83 |
| **AB3** | 0.14 | 0.43 | 12.96 | 6.55 | 1.38 | 1.22 | 1.31 | 2.33 |
|  |  |  |  |  |  |  |  |  |
| **ACO1** | 0.65 | 0.94 | 4.33 | 2.59 | 1.26 | 1.20 | 2.22 | 2.00 |
| **ACO2** | 0.30 | 0.44 | 6.28 | 5.81 | 1.42 | 1.42 | 1.33 | 1.80 |
| **ACO3** | 0.31 | 0.44 | 9 | 6.7 | 1.81 | 1.43 | 1.52 | 2.08 |
|  |  |  |  |  |  |  |  |  |
| **AC1** | 0.31 | 0.38 | 6.58 | 6.61 | 1.29 | 1.29 | 1.60 | 1.95 |
| **AC2** | 0.37 | 0.48 | 5.74 | 4.74 | 1.32 | 1.17 | 1.62 | 1.93 |
| **AC3** | 0.30 | 0.46 | 4.85 | 4.2 | 1.07 | 1.23 | 1.34 | 1.58 |
|  |  |  |  |  |  |  |  |  |
| **AMLH1** | 0.37 | 0.31 | 8.01 | 7.57 | 1.25 | 1.25 | 2.35 | 1.90 |
| **AMLH2** | 0.27 | 0.21 | 8.22 | 10.5 | 1.19 | 1.36 | 1.84 | 1.65 |
| **AMLH3** | 0.16 | 0.23 | 12.6 | 9.27 | 1.55 | 1.33 | 1.33 | 1.58 |
|  |  |  |  |  |  |  |  |  |
| **AODH1** | 0.51 | 0.65 | 5.24 | 3.85 | 1.58 | 1.54 | 1.70 | 1.63 |
| **AODH2** | 0.54 | 0.59 | 5.68 | 3.29 | 1.61 | 1.25 | 1.91 | 1.63 |
| **AODH3** | 0.53 | 1.44 | 3.54 | 1.9 | 1.47 | 1.46 | 1.28 | 1.63 |
|  |  |  |  |  |  |  |  |  |
| **AK1** | 0.51 | 1.48 | 15.55 | 4.2 | 1.56 | 1.11 | 5.05 | 5.59 |
| **AK2** | 0.37 | 1.15 | 15.71 | 7.88 | 1.57 | 1.40 | 3.74 | 6.45 |
| **AK3** | 0.77 | 0.58 | 11.59 | 10.21 | 1.50 | 1.36 | 5.98 | 4.38 |
|  |  |  |  |  |  |  |  |  |
| **ACDT1** | 0.48 | 1.00 | 9.54 | 6.27 | 1.05 | 1.04 | 4.33 | 6.01 |
| **ACDT2** | 0.71 | 0.59 | 8.46 | 6.91 | 0.85 | 1.16 | 7.03 | 3.51 |
| **ACDT3** | 0.82 | 0.68 | 7.52 | 6.12 | 0.97 | 0.76 | 6.38 | 5.48 |
|  |  |  |  |  |  |  |  |  |
| **Soual_01_1** | 4.48 |  | 0.75 |  | 0.57 |  | 5.89 |  |
| **Soual_01_2** | 5.12 |  | 0.56 |  | 0.32 |  | 8.95 |  |
| **Soual_01_3** | 5.34 |  | 0.49 |  | 0.27 |  | 9.69 |  |
|  |  |  |  |  |  |  |  |  |
| **Soual_02_1** | 3.47 |  | 1.82 |  | 1.57 |  | 4.03 |  |
| **Soual_02_2** | 3.50 |  | 1.88 |  | 1.62 |  | 4.08 |  |
| **Soual_02_3** | 3.25 |  | 1.79 |  | 1.66 |  | 3.51 |  |
|  |  |  |  |  |  |  |  |  |
| **ART (ng/ml)** | 2.72 |  |  |  |  |  |  |  |

a.ART(tea)_IC_50_ = C_ART x (IC_50_/C_Tea)
